# Natural Enzyme‐Loaded Polymeric Stealth Coating‐Armed Engineered Probiotics by Disrupting Tumor Lactate Homeostasis to Synergistic Metabolism‐Immuno‐Enzyme Dynamic Therapy

**DOI:** 10.1002/advs.202417172

**Published:** 2025-02-28

**Authors:** Liuzhou Mao, Bahriman Xarpidin, Rui Shi, Yuting Lin, Haohua Hu, Caisheng Wu, Zheng Luo, Yun‐Long Wu

**Affiliations:** ^1^ State Key Laboratory of Cellular Stress Biology Fujian Provincial Key Laboratory of Innovative Drug Target Research School of Pharmaceutical Sciences Xiamen University Xiamen 361102 China

**Keywords:** engineered probiotics, enzyme therapy, immunoregulation, lactate metabolism, polymeric stealth coating

## Abstract

Reducing L‐lactate levels in tumors is crucial for alleviating immunosuppression and enhancing treatment efficacy. Recently, bacteria have great potential in improving lactate levels in the tumor microenvironment due to their physiological properties, tumor tropism, and immunogenicity. However, developing bacterial‐based lactate regulation platforms is still facing great challenges due to bacterial modification impacts on activity, macrophage phagocytosis, and complex tumor microenvironment. Herein, an engineered *Lactobacillus acidophilus* (LH@LA) is developed, armed with a polymeric stealth coating that co‐loads lactate oxidase (LOx) and horseradish peroxidase (HRP). This coating protects bacteria from macrophage phagocytosis, maintaining their activity for deep tumor drug delivery. And then, LOx and HRP can consume much L‐lactate in the tumor site through an enzyme cascade reaction to improve the immunosuppressive environment, while causing oxidative stress and reduced ATP supply, thereby reversing AKT‐mTOR metabolic pathway activation to inhibit tumor growth. More interestingly, LA not only acts as a natural enzyme's carrier, but also induces anti‐inflammatory M2 macrophages to polarize into pro‐inflammatory M1 macrophages by secreting D‐lactate, enhancing antitumor immunotherapy. This engineered probiotic design provides a new idea for building a safe and efficient live bacteria delivery platform, providing a reference for developing cancer treatment strategies with clinical translation prospects.

## Graphical abstract

Engineered *Lactobacillus acidophilus* (LH@LA) is developed via in situ polymerization to co‐load lactate oxidase (LOx) and horseradish peroxidase (HRP). This system preserves LA activity, evades macrophage phagocytosis, and synergizes LA metabolism with enzyme cascades to enhance immunity, reduce tumor ATP, suppress PD‐L1 expression and AKT‐mTOR activation, thereby effectively eradicating cancer cells.

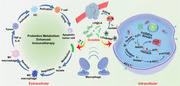

## 1 Introduction

Lactate, as one of the abnormal metabolites of tumor cells, plays a key role in the tumor immunosuppressive microenvironment, drug resistance, and tumor recurrence and metastasis.^[^ ^1^ ^]^ It usually has two optical isomers: L‐lactate and D‐lactate. The former is the main product of intracellular glycolysis, widely present in tumor tissues, and is a key factor leading to tumor immunosuppressive microenvironment. The latter is mainly produced by intestinal probiotics, which can regulate the repolarization of anti‐inflammatory M2 macrophages to pro‐inflammatory M1 macrophages, and has the effect of reshaping the tumor immunosuppressive microenvironment.^[^ ^2^ ^]^ In addition, it is reported that L‐lactate accumulation will induce tumor‐associated macrophages to polarize to the anti‐inflammatory M2 type, inhibit the secretion of pro‐inflammatory factors, and regulate the expression of various cytokines and chemokines to promote the repair and regeneration of tumor tissues, thereby reducing the antitumor effect.^[^ ^3^ ^]^ In addition, excessive L‐lactate will upregulate the expression of programmed cell death ligand 1 (PD‐L1) protein in tumor cells and activate the AKT‐mTOR metabolic pathway, thereby reducing the proliferation and T cell cytotoxicity and promoting the malignant development of tumors.^[^ ^4^ ^]^ Therefore, effective regulation of D/L‐lactate levels has become a promising treatment method for improving the tumor immunosuppressive microenvironment and enhancing the effect of tumor treatment.^[^ ^5^ ^]^

Bacteria, as long‐term symbionts of humans, are widely present in various parts such as the skin, mouth, and intestines, and participate in physiological processes such as metabolism, immunity and the gut‐brain axis, which shows great potential for regulating lactate levels in tumor tissues.^[^ ^6^ ^]^ In recent years, some bacteria such as *Shewanella oneidensis* MR‐1 have shown efficient lactate consumption ability, which can not only improve the antitumor effect by improving the tumor microenvironment but also serve as a bridge to combine other tumor therapies.^[^ ^7^ ^]^ However, relying solely on the lactate metabolism ability of bacteria is not efficient. Through physical adsorption (such as electrostatic effect), chemical modification, genetic engineering, etc., bacterial carriers can combine nanoparticles, metal–organic frameworks, chemotherapy drugs, nanozymes, natural enzymes (lactate oxidase), etc., to more efficiently accelerate the consumption of lactate in tumor tissues, thereby synergistically strengthening chemotherapy, radiotherapy, catalytic therapy, and immunotherapy, etc.^[^ ^7^, ^8^ ^]^ For example, Liu et al. designed a *Lactococcus lactis*‐based drug delivery vehicle that expresses a fusion protein of Flt3L and OX40 ligand. Intratumoral injection of FOLactis was shown to directly kill cancer cells through cell lysis and induce a potent antitumor immune response.^[^ ^9^ ^]^ Arpaia et al. engineered a modified strain of the probiotic Escherichia coli Nissle 1917 as an antitumor vaccine platform. By encoding tumor neoantigens into these engineered bacteria, they utilized the bacteria to deliver tumor neoantigens, thereby training the immune system to target and attack cancer cells expressing the same tumor neoantigens.^[^ ^10^ ^]^ Given the unique characteristics of bacteria such as hypoxic colonization, immunogenicity, and easy modification, bacterial‐based nanomedicine has shown great potential in the field of tumor treatment.^[^ ^11^ ^]^ However, most of the bacteria currently used to metabolize lactate are non‐native bacteria in the body, and there are worrying safety risks. Intestinal probiotics are active microorganisms that are beneficial to host health.^[^ ^12^ ^]^ They can prevent and treat diseases by inhibiting the growth of harmful bacteria, activating immune cells, and secreting beneficial metabolites. For example, *Lactobacillus acidophilus* (LA) can produce D‐lactate with immunomodulatory function.^[^ ^13^ ^]^ We speculate that equipping LA with lactate oxidase would not only enable efficient consumption of L‐lactate, but also promote anti‐inflammatory M1 macrophage polarization via the bacterial metabolite D‐lactate, which would effectively enhance tumor immunotherapy.^[^ ^2^ ^]^ However, current bacterial modification methods usually make it difficult to efficiently load natural enzymes while maintaining bacterial activity, and bacterial carriers are generally easily phagocytosed and cleared by macrophages in the body, thereby reducing their tumor treatment effects. Therefore, developing a method that can ensure bacterial activity and effectively load lactate oxidase while evading macrophage phagocytosis is crucial for building a safe and efficient antitumor live bacterial‐based platform.

**Scheme 1:**
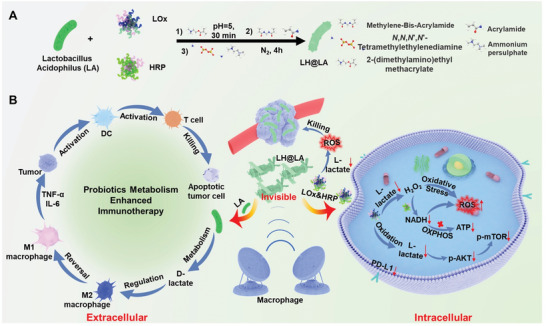
Synthesis of LH@LA and its antitumor mechanism. A) LH@LA was synthesized by electrostatic adsorption and in situ free radical polymerization. B) LH@LA mechanism of action in tumor cells and tumor microenvironment.

Here, we developed an engineered LA (LH@LA) armed with a polymer coating loaded with natural enzymes LOx and HRP (**Scheme** **1**). Double bonds were modified on the bacterial, lactate oxidase (LOx), and horseradish peroxidase (HRP) surfaces by electrostatic action, and then polymerized on the bacterial surface by in situ polymerization, thereby obtaining LA wrapped in a polymer coating co‐loaded with LOx and HRP.^[^ ^14^ ^]^ This method can effectively retain LA activity and protect it from being phagocytosed by macrophages, thereby effectively transporting the cargo to the deep part of the tumor to exert its effect.^[^ ^15^ ^]^ The polymer loaded with LOx and HRP can not only efficiently consume L‐lactate in the tumor microenvironment through enzyme cascade reactions to generate many reactive oxygen species (ROS), but can also fall off from the bacterial surface in the tumor microenvironment and enter cancer cells, causing oxidative stress in tumor cells, thereby damaging the mitochondria of cancer cells. This L‐lactate consumption from the inside out greatly reduces the L‐lactate level of tumor tissue, improves the tumor immunosuppressive microenvironment while reducing the adenosine triphosphate (ATP) supply, and can effectively inhibit the expression of PD‐L1 in tumor cells and the activation of the AKT‐mTOR metabolic pathway, thereby enhancing antitumor effect. What is more interesting is that LA not only acts as a bacterial carrier but also produces D‐lactate, which induces the polarization of anti‐inflammatory M2 macrophages to pro‐inflammatory M1 macrophages, increasing the release of inflammatory factors, thus activating and strengthening the immune system to effectively eliminate cancer cells. This preparation method provides a new idea for the development of a safe and efficient live bacterial delivery platform, and this treatment strategy combining metabolic‐immune‐enzyme dynamic therapy provides a promising clinical cancer treatment method with good translational prospects.

## 2 Results and Discussion

### 2.1 Characterization and Functional Verification of LH@LA

**Figure 1:**
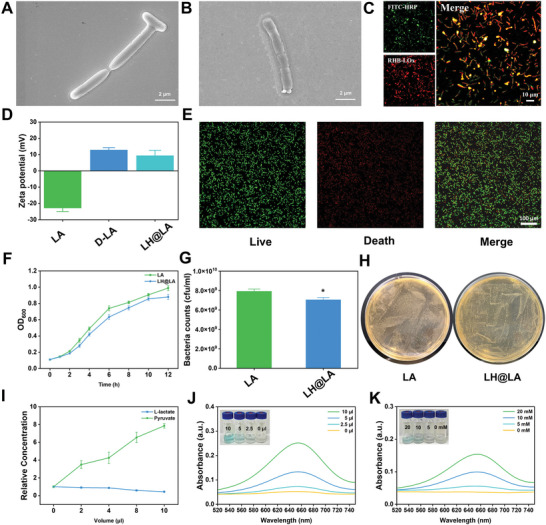
Preparation and characterization of LH@LA. SEM images of A) LA (Scale bar: 2 µm) and B) LH@LA (Scale bar: 2 µm). C) Fluorescence imaging of LH@LA synthesized from FITC‐labeled HRP and Rhodamine B‐labeled LOx (Scale bar: 10 µm). D) LA, D‐LA, and LH@LA potential diagrams (*n* = 3). E) Fluorescence imaging of bacteria with live/dead staining of LH@LA (Scale bar: 100 µm). F) OD_600_ values of LA and LH@LA at different time points. G) Bacterial counts were quantified for LA and LH@LA at 12 h after incubation in the medium. H) Images of LA and LH@LA at 12 h incubation in MRS (De Man, Rogosa and Sharpe) agarose solid medium. I) Relative concentrations of lactate and pyruvate in solution after catalysis by different volumes of LH@LA in L‐lactate solution. UV–vis absorption spectra of TMB solutions at J) different LOx@LA concentrations (OD_600_ = 0.2) and K) different L‐lactate concentrations.

LH@LA was prepared by a simple one‐step in situ polymerization method based on the previous research work of our research group.^[^ ^16^ ^]^ Prior to synthesizing LH@LA, we explored various ratios of LOx to HRP, taking into account both economic efficiency and cytotoxicity toward tumor cells. Ultimately, we opted to use 1 mg each of LOx and HRP for the synthesis of LH@LA (Figure S1, Supporting Information). As shown in **Figure** **1**A, the surface of the unmodified LA is relatively smooth, while the surface of the modified LH@LA is granular (Figure 1B), indicating that the bacterial surface can be effectively modified by this method. To further verify whether the enzyme can be loaded into the bacterial surface coating, we added FITC‐labeled HRP (FITC‐HRP) and Rhodamine B‐labeled LOx (RHB‐LOx) to the reaction system. As shown in Figure 1C and Figure S2 (Supporting Information), FITC‐HRP, and RHB‐LOx can be effectively co‐loaded onto the surface of LA through our synthesis method, with an enzyme loading capacity reaching 74.15%. In addition, the zeta potential analysis of LA, 2‐(dimethylamino) ethyl methacrylate (2‐DM) modified LA (defined as D‐LA), and LH@LA revealed that the potential of D‐LA is significantly enhanced due to the positive charge introduced by 2‐DM compared to LA. The potential of LH@LA is slightly reduced relative to D‐LA, likely due to the loading of the natural enzymes LOx and HRP (Figure 1D). All the above results indicated that the natural enzymes were successfully loaded onto the surface of LA. To verify the effect of the synthesis method on the activity of the bacteria, we further evaluated the survival and growth of the bacteria. According to the live/dead staining analysis of bacteria (Figure 1E), the area of green fluorescence was significantly higher than that of red fluorescence (green represents live bacteria, red represents dead bacteria), indicating that the method had less damage to bacteria. Through further analysis of bacterial proliferation (Figure 1F, H), the results showed that the modified bacteria still had a similar growth trend as the unmodified bacteria, indicating that the method had little damage to the bacteria. Then, the enzyme activity of LH@LA was studied to preliminarily explore its potential antitumor ability. The lactate detection kit and the pyruvic acid detection kit were used to evaluate the activity of LOx in the bacterial coating of LH@LA. As shown in Figure 1I, with the increase of the amount of LH@LA, the L‐lactate in the solution was gradually catalyzed by LOx to generate pyruvic acid.

In addition, the reaction efficiency of LOx in the LOx@LA bacterial coating was also evaluated by the tetramethylbenzidine (TMB) colorimetric method. LOx@LA showed good enzyme catalytic efficiency and could effectively oxidize TMB into blue ox‐TMB, as shown by the enhanced intensity of its characteristic absorption peak (652 nm), and its catalytic effect increased with the increase of LOx@LA or L‐lactate concentration (Figure 1J,K). The above experimental results show that this synthesis method does not destroy the activity of LA and natural enzymes, providing a guarantee for the subsequent application of tumor therapy.

Subsequently, to verify the long‐term stability of LH@LA after synthesis when stored in PBS solution, we first captured images of the morphology of LH@LA at different time points using scanning electron microscopy (SEM) and analyzed the zeta potential of LH@LA using a nano‐sizer and zeta potential analyzer. We found that after 5 days of storage, there were no significant changes in morphology or a notable decrease in zeta potential values (Figures S3 and S4, Supporting Information). Furthermore, we confirmed the enzyme activity of LOx@LA at various time points through a TMB colorimetric assay, which also showed that enzyme activity was well preserved (Figure S5, Supporting Information). Additionally, we cultured LH@LA samples stored for different periods on MRS solid medium and observed that the activity of LA was well maintained (Figure S6, Supporting Information). These experimental results demonstrate that all physicochemical properties of LH@LA can be effectively preserved for up to 5 days, ensuring the orderly progression of experiments.

### 2.2 Evaluation of the Antitumor Model of LH@LA In Vitro

**Figure 2:**
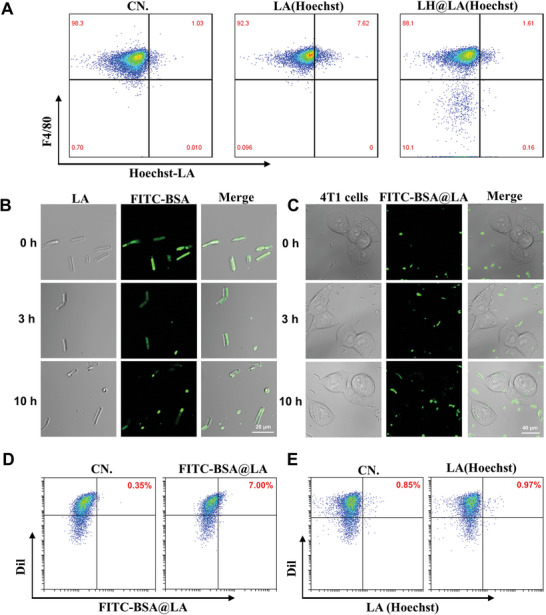
Evaluation of the antitumor effect model of LH@LA in vitro. A) Quantitative analysis of the different uptake behaviors of LA and LH@LA by RAW 264.7 cells using flow cytometry. B) The fluorescence imaging of FITC‐labeled BSA@LA at 0, 3, 10 h (Scale bar: 20 µm). C) The uptake of FITC‐labeled BSA by 4T1 cells at 0 3, 10 h was observed by fluorescence imaging (Scale bar: 40 µm). D) Quantitative analysis of 4T1 cell uptake of FITC‐BSA@LA using flow cytometry. E) Quantitative analysis of 4T1 cell uptake of Hoechst‐LA using flow cytometry.

To demonstrate that modified LA can effectively avoid phagocytosis by macrophages and thus exert LA metabolism. Fluorescein Isothiocyanate (FITC)‐labeled LA and bovine albumin (BSA)@FITC‐labeled LA were added into confocal cell culture dishes coated with macrophage RAW 264.7, respectively. As shown in **Figures** **2**A and S7 (Supporting Information), the Hoechst‐labeled LA were taken up by RAW 264.7 cells after 6 h. In contrast, the LH@Hoechst‐labeled LA, modified using our synthetic method, exhibited a significantly reduced phagocytic uptake by macrophages. These experimental results demonstrated that the LA modified by a simple one‐step in situ polymerization method can effectively help the LA to escape from macrophage phagocytosis.

In addition, to evaluate the stability of the polymeric coating on the LH@LA surface and the antitumor model of LH@LA, FITC‐labeled BSA was used as a model protein to verify the residence time of the polymeric coating prepared by the synthesis method on the bacterial surface. As shown in Figure 2B, the polymeric coating loaded with FITC‐labeled BSA did begin to fall off from the bacterial surface until 10 h and was taken up by cancer cells in large quantities (Figure 2C). To further validate the behavior of LH@LA in 4T1 cells, quantitative studies were conducted on the uptake of FITC‐BSA@LA and Hoechst‐labeled LA by 4T1 cells using flow cytometry. As shown in Figure 2D,E, the uptake of FITC‐BSA@LA by Dil‐labeled 4T1 cells reached ≈ 7% after 10 h, while there was minimal uptake of Hoechst‐labeled LA. This evidence strongly supports that the action mechanism of LH@LA in 4T1 cells involves LOx and HRP entering tumor cells to exert antitumor effects, whereas LA remains in the tumor microenvironment to play its role. The above experimental results fully demonstrate the effectiveness of the transformation method adopted in this study and its advantages in constructing a live bacteria delivery platform.

### 2.3 Investigation of the Antitumor Effect and Mechanism of LH@LA

**Figure 3:**
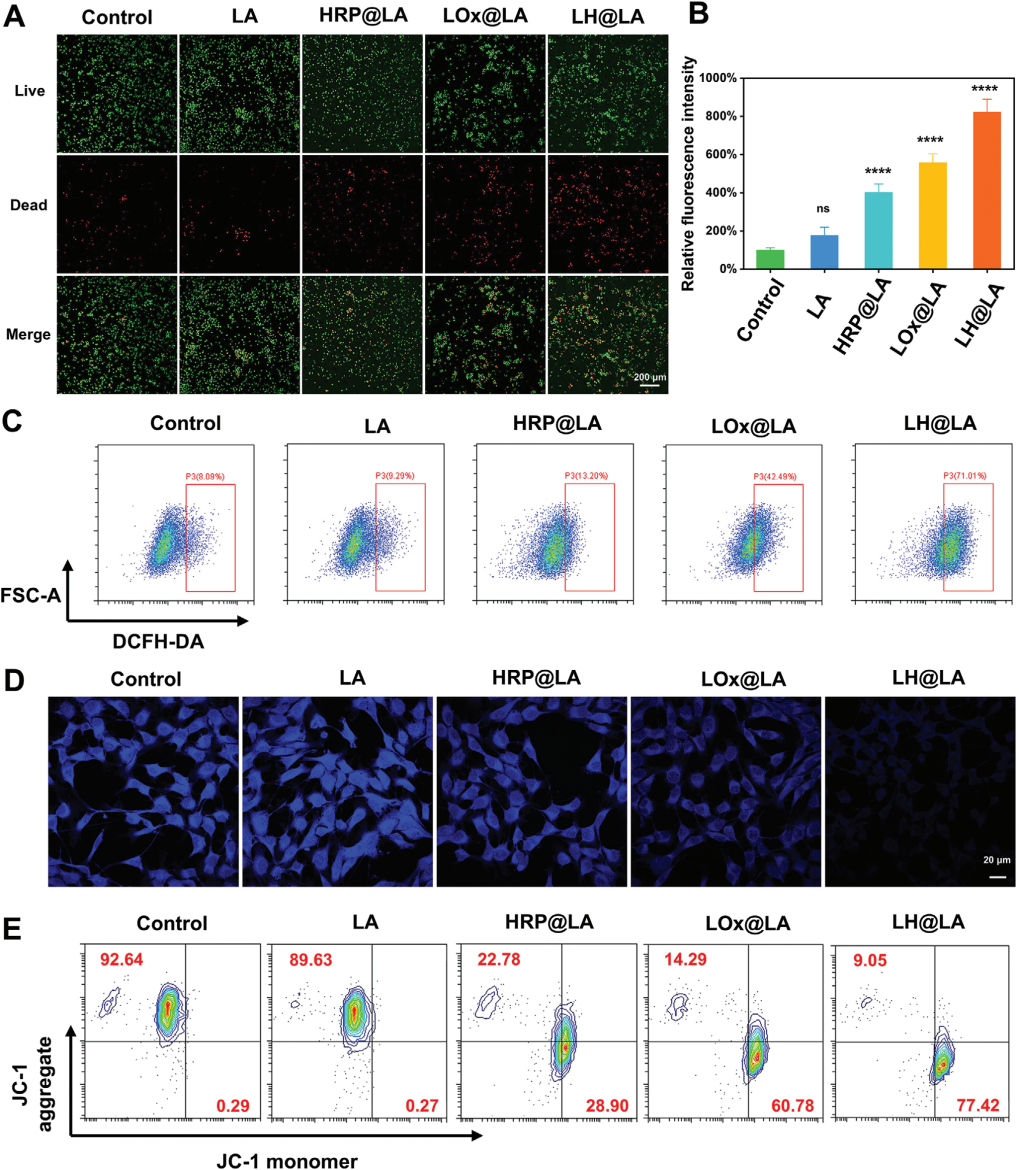
Investigation of the antitumor effect and mechanism of LH@LA. A) Fluorescence imaging of 4T1 cells with Live/Dead staining after treatment with different materials (Scale bar: 200 µm). B) Fluorescence quantification analysis of red fluorescence (dead cells) in Figure 3A. C) Flow cytometry to detect ROS production in 4T1 cells treated with different materials. D) GSH fluorescence imaging of 4T1 after treatment with different materials (Scale bar: 20 µm). E) Flow cytometry detection of MMP changes in 4T1 after treatment with different materials.

According to the experimental results in the previous section, the mode of antitumor effect exerted by LH@LA is due to the catalytic action of the natural enzyme inside the cell and the metabolic action of LA outside the cell. LH@LA can consume a large amount of L‐lactate in tumor cells from the inside out through an enzyme cascade reaction and generate highly toxic free radicals, thereby causing oxidative damage to 4T1 cells, HeLa cells, and MCF‐7 cells (**Figure** **3**A,B; Figure S8, Supporting Information), showing the strongest cytotoxicity. More encouragingly, using L929 as a representative model of normal cells, we administered LH@LA following the same experimental protocol and performed live/dead staining. The results showed that LH@LA did not exhibit significant cytotoxicity toward normal cells (Figure S9, Supporting Information). By analyzing the apoptosis of cells by flow cytometry and fluorescence imaging (Figures S10 and S11, Supporting Information), the LH@LA group still showed the most apoptotic cells, indicating that it has good antitumor potential. In addition, to investigate the principle of LH@LA‐induced apoptosis in tumor cells. First, it is well known that high levels of ROS can directly disrupt the oxidative balance in tumor cells, thereby inducing tumor cell apoptosis. As shown by confocal laser scanning microscope (CLSM) fluorescence imaging, tumor cells after the treatment of LH@LA produced the strongest green fluorescence, indicating the highest level of ROS. In contrast, tumor cells after the action of HRP@LA and LOx@LA had weaker fluorescence intensity (Figure S12, Supporting Information). In addition, flow cytometry analysis showed that 71.1% of the tumor cells after the treatment with LH@LA produced intense oxidative stress, this data was ≈ nine‐fold higher than that of the control group (Figure 3C).

Furthermore, it has been reported that H_2_O_2_ synergistically with HRP can induce a decrease of intracellular glutathione (GSH) and effectively inhibit the electron transport chain of nicotinamide adenine dinucleotide (NADH), leading to strong oxidative stress in mitochondria. As observed by CLSM, LH@LA resulted in a significant decrease in GSH concentration due to the synergistic effect between LOx‐catalyzed H_2_O_2_ generated from L‐lactate and HRP. In contrast, LOx@LA and HRP@LA had less effect on intracellular GSH concentration in tumor cells due to the lack of a cascade reaction system (Figure 3D). Moreover, we quantitatively and qualitatively examined the mitochondrial membrane potential (MMP) changes in 4T1 cells after treatment with different materials by flow cytometry and fluorescence experiments. Flow cytometry results demonstrated that LH@LA could lead to a significant decrease in MMP occurrence in 77.42% of 4T1 cells, this number was about 266‐fold higher than the control group (Figure 3E). In addition, the results obtained from CLSM fluorescence imaging were consistent with flow cytometry (Figure S13, Supporting Information). The above experimental results can fully explain the mechanism of LH@LA‐induced apoptosis in tumor cells, which is caused by the LOx/HRP enzyme cascade reaction system catalyzing the production of large amounts of ROS from lactate and destroying the oxidative stress defense system of the tumor cells, which causes a decrease in MMP of the cells.

### 2.4 Study of LH@LA for Improving the Tumor Immune Microenvironment

**Figure 4:**
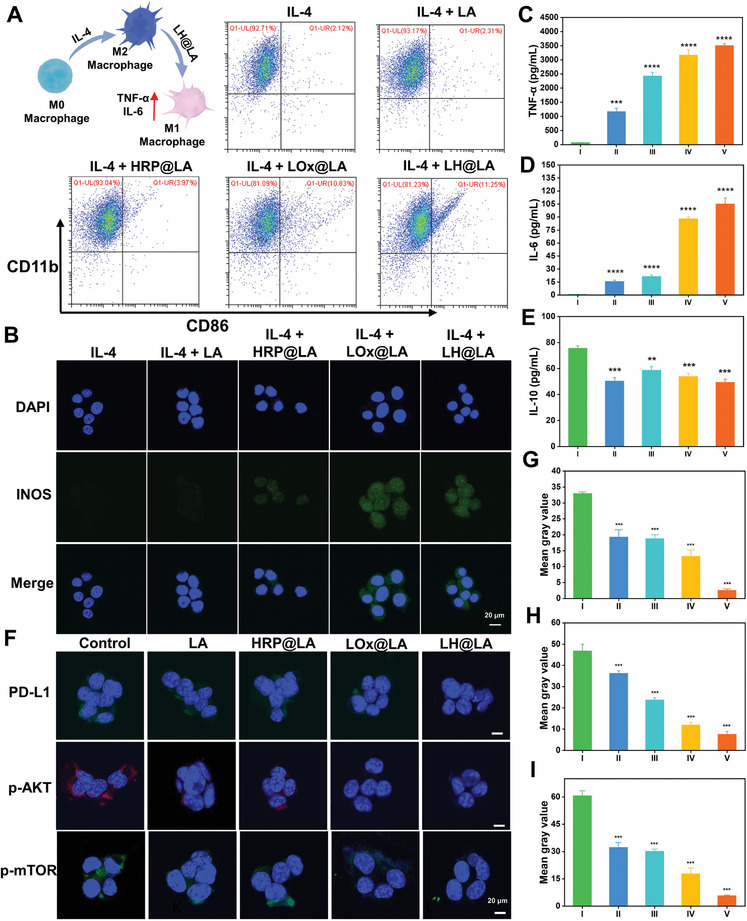
Study of LH@LA for improving the tumor immune microenvironment. A) Flow cytometry to detect the ability of different groups of materials to induce repolarization of M2 macrophages into M1 macrophages. B) INOS fluorescence imaging of RAW 264.7 macrophages treated with different administration methods (Scale bar: 20 µm). Elisa assay kit to detect C) TNF‐α, D) IL‐6, E) IL‐10 concentration in the supernatant of RAW 264.7 macrophages after treatment with different administration groups (*n* = 3) (I: IL‐4, II: IL‐4+LA, III: IL‐4+HRP@LA, IV: IL‐4+LOx@LA, V: IL‐4+LH@LA). F) Fluorescence imaging of PD‐L1, phosphorylated AKT, and phosphorylated mTOR expressed in 4T1 cells after treatment with different administration groups (Scale bar: 20 µm). Fluorescence quantification plots of G) PD‐L1, H) phosphorylated AKT, and I) phosphorylated mTOR expressed in 4T1 cells after treatment with different administration groups (*n* = 3) (I: Control, II: LA, III: HRP@LA, IV: LOx@LA, V: LH@LA). Data are presented as the mean ± standard deviation (SD). Statistical significance was calculated by t‐test for comparison between two groups. ns: no significant, ^**^ *p* ≤ 0.01, ^***^ *p* ≤ 0.001, ^****^ *p* ≤ 0.0001.

Stimulating the body's own immune system plays an important role in tumor immunotherapy.^[^ ^17^ ^]^ The ability of LH@LA to induce repolarization of tumor‐associated macrophages (TAM) anti‐inflammatory M2 macrophages into pro‐inflammatory M1 macrophages was investigated by flow cytometry and CLSM. As shown in **Figure** **4**A, ≈11.25% of M2 macrophages in the IL‐4 (+ LH@LA group repolarized to M1 macrophages, which was ≈five times that of the IL‐4 group. Subsequently, the RAW 264.7 macrophages treated with IL‐4 and different drugs were stained with fluorescently labeled INOS (M1 macrophage antibody) and ARG‐1 (M2 macrophage antibody). CLSM observation showed that the green fluorescence intensity of INOS on the surface of RAW 264.7 cells treated with IL‐4+LH@LA was the highest, while the red fluorescence intensity of ARG‐1 was the lowest, indicating that it can effectively reverse M2 macrophages into M1 macrophages (Figure 4B; Figure S14, Supporting Information). The cytokine concentrations in the supernatants of macrophages treated with different administration groups were then quantified by means of ELISA assay kits. As shown in Figure 4C–E, in IL‐4 + LH@LA group, the pro‐inflammatory factors tumor necrosis factor‐α (TNF‐α) and Interleukin‐6 (IL‐6) were significantly higher than the other groups, whereas the anti‐inflammatory factor IL‐10 was lower compared to the IL‐4 group.

In addition, the expression levels of PD‐L1, phospho‐AKT (p‐AKT), and phospho‐mTOR (p‐mTOR) on the surface of tumor cells treated with different materials were reflected by immunofluorescence labeling. As shown in Figure 4F, the PD‐L1, p‐AKT, and p‐mTOR expression levels of the tumor cells treated by LH@LA all showed a significant decline. The quantitative fluorescence data can also show that the cellular PD‐L1, p‐AKT, and p‐mTOR expression levels in the LH@LA treatment group are consistent with the conclusion of the fluorescence experiment (Figure 4G–I). The above experimental results show that LH@LA helps to reduce the expression of PD‐L1 in tumor cells and inhibit tumor metabolism, thereby inhibiting tumor immune escape, proliferation, and migration, and enhancing tumor immunotherapy.

### 2.5 Mechanistic Studies of LH@LA‐Induced Macrophage Repolarization and Impact on Tumor Metabolism

**Figure 5:**
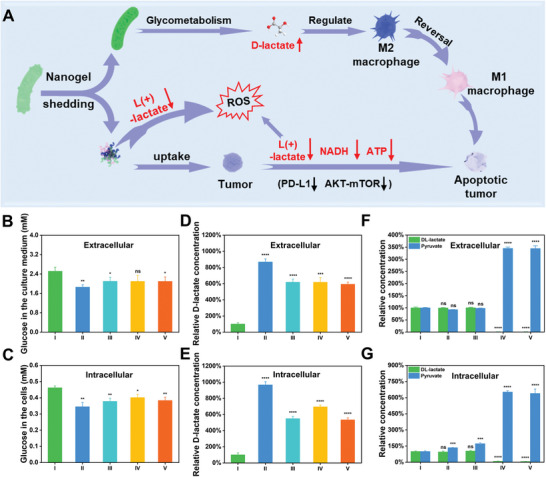
Mechanistic studies of LH@LA‐induced macrophage repolarization and impact on tumor metabolism. A) A schematic diagram of probiotic metabolism and enzyme catalysis of LH@LA. A kit was used to detect the glucose concentration B) in the supernatant of 4T1 cells and C) in the 4T1 cells after treatment in the different administered groups (*n* = 3). A kit was used to detect the concentration of D‐ lactate D) in the supernatant of 4T1 cells and E) in the 4T1 cells after treatment in the different administration groups (*n* = 3). Total lactate concentration and pyruvate concentration F) in the supernatant of 4T1 cells and G) in the 4T1 cells after treatment in different dosing groups were measured by kits (*n* = 3). (I: Control, II: LA, III: HRP@LA, IV: LOx@LA, V: LH@LA). Statistical significance was calculated by t‐test for comparison between the two groups. (I: Control, II: LA, III: HRP@LA, IV: LOx@LA, V: LH@LA). ns: no significant, ^*^ *p* ≤ 0.05, ^**^ *p* ≤ 0.01, ^***^ *p* ≤ 0.001, ^****^ *p* ≤ 0.0001.

First, based on the previous sections, the action mechanism of LH@LA may be as shown in **Figure** **5**A. Since LA metabolism to produce D‐lactate requires nutrient support in the culture medium, LA may have the ability to compete with tumor cells for nutrients. Thus, the glucose concentration in the tumor cells and the culture medium was measured by using a glucose concentration assay kit to reflect the metabolic activity of LA in the culture medium. As shown in Figure 5B,C, although the glucose concentration in the LH@LA group was elevated compared to the LA group, LH@LA still showed a significant decrease in glucose concentration compared to the control group. And the changing trend of glucose concentration in the tumor cells was like that in the culture medium. In addition, the D‐lactate content inside and outside the tumor cells was also measured by a D‐lactate assay kit, to verify whether the repolarization of M2 macrophages into M1 macrophages was induced due to D‐lactate secreted by LA. As shown in Figure 5D,E, compared with the control group, the relative concentrations of extracellular and intracellular D‐lactate in the LH@LA group increased to more than about 6 and 5 times that of the control group, respectively. To verify the effect of LH@LA on the metabolism of tumor cells, we then examined the total lactate concentration and pyruvate concentration intracellularly and extracellularly in the tumor cells. As shown in Figure 5F, using control as a comparison, the relative extracellular lactate concentration in the LH@LA group decreased to less than one‐tenth of that in the control group. In contrast, pyruvate, as a product of LOx‐catalysed L‐lactate catabolism, the concentration of pyruvate was elevated to about 3‐fold higher than the control group. Similarly, in comparison to the control group, the lactate and pyruvate concentrations in the tumor cells decreased by 14‐fold and increased by about 6‐fold, respectively (Figure 5G). Therefore, it can be concluded that the inhibition of AKT‐mTOR metabolic pathway activation in tumor cells should be attributed to the LOx‐catalyzed L‐lactate catabolism.

In addition, tumor cells can alternatively use glycolysis and oxidative phosphorylation (OXPHOS) as sources of ATP production. And the reduced forms of nicotinamide adenine dinucleotide (NADH) are essential cofactors for OXPHOS. As shown in Figure S15 (Supporting Information), as it was obtained by the NADH/NAD^+^ assay kit, the NADH concentration in the LH@LA group decreased to about three‐fifths of the control group. In addition, the reduction of the NADH consequently affects the OXPHOS process in the mitochondria of tumor cells, thus leading to the inhibition of their ATP synthesis (Figure S16, Supporting Information).

### 2.6 Study of Tumor Targeting and Antitumor Efficacy of LH@LA In Vivo

**Figure 6:**
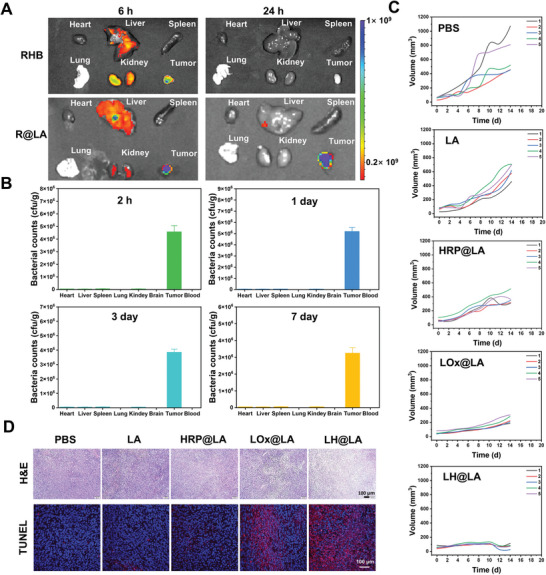
Study of tumor targeting and antitumor efficacy of LH@LA in vivo. A) Fluorescence imaging of RHB and R@LA in various organs and tumor sites at 6 and 24 h of intratumorally injection in situ. B) Quantitative distribution plots of LA in various organs, brain, tumor, and blood at 2 h, 1 day, 3 days and 7 days of intratumoural injection in situ (*n* = 3). C) Quantitative plot of tumor volume change over 14 days after treatment with different groups of materials (*n* = 5). D) H&E staining (scale bar: 100 µm) and TUNEL fluorescence staining (scale bar: 100 µm) of tumor tissue sections after different drug treatments.

According to the above experimental results, we evaluated the antitumor ability of LH@LA by establishing a mouse 4T1 tumor model and administering it over several doses. First, the tumor‐targeting ability of LH@LA in vivo was evaluated. To verify the tumor‐targeting ability of LA, we labeled the bacterial vector (R@LA) with rhodamine B (RHB) for tracking. The in vivo distribution of RHB and R@LA after intratumoral injection was detected by the Caliper IVIS Lumina II imaging system. As shown in **Figure** **6**A, after RHB and R@LA were injected intratumorally in mice separately, the fluorescent signals of the RHB‐injected group had been mostly metabolized at 6 h, whereas the fluorescent signals of the R@LA‐injected group were mainly concentrated in the tumor site. Moreover, the fluorescence signal of R@LA was significantly enriched in the tumor site 24 h after injection, while RHB injected alone was completely metabolized at 24 h and did not show a significant fluorescence signal. Subsequently, to demonstrate the selective colonization of LA in the tumor, tumor tissues, major organs, and blood were harvested, ground, and centrifuged to take the supernatant in MRS medium and quantified for bacterial counts at 2 h, 1, 3, and 7 days after intratumoral injection of LA. As shown in Figure 6B, there was a large amount of LA colonization in the tumor area and less in other major organs, further indicating that LA can selectively colonize the tumor site. Additionally, to distinguish between the bacteria present in the tumor (tumor‐associated bacteria) and LA, we performed plating experiments using representative Gram‐negative bacteria (*Escherichia coli*) and Gram‐positive bacteria (*Staphylococcus aureus*). These bacteria were plated on both MRS agar and LB agar. We observed that neither *E. coli* nor *S. aureus* could grow on MRS agar, indicating that LA can grow in MRS medium, while not all bacterial species can do so (Figure S17, Supporting Information).

Thereafter, LH@LA in vivo antitumor potential in a subcutaneous mouse 4T1 tumor model was evaluated. When the tumor volume reached a certain size, mice were randomly divided into five groups (*n* = 5): 1) PBS; 2) LA;3) HRP@LA; 4) LOx@LA; 5) LH@LA. Subsequently, intratumoral injections of PBS, LA, HRP@LA, LOx@LA, and LH@LA were administered to mice. And tumor size and body weight of mice were measured every 2 days. As shown in Figure 6C and Figure S18 (Supporting Information), tumor growth was rapid in the PBS, LA, and HRP@LA groups of mice, suggesting that injection of PBS, LA, or HRP@LA alone had negligible effect on tumor growth. In contrast, the tumor growth of the LOx@LA group was slightly inhibited, which might be caused by the reaction of LOx with L‐lactate to produce ROS. Most excitingly, the tumor growth in the LH@LA group was significantly inhibited, which was caused by the combined effect of LOx and HRP enzyme cascade reaction system and LA metabolism, and it was consistent with the conclusions obtained from our cellular experimental analysis. Furthermore, it was also evident by TUNEL staining and tumor tissue section H&E staining that significant apoptosis occurred in the tumor tissues of the LH@LA group (Figure 6D). Moreover, tumor tissue image and H&E staining of major organ sections showed that LH@LA could effectively inhibit tumor growth while causing no significant damage to major organs (Figure S19, Supporting Information). Similarly, analysis of blood Alkaline phosphatase (ALP), Albumin (ALB), Aspartate transaminase (AST), and Alanine aminotransferase (ALT) of mice after administration of LH@LA shows that LH@LA is safe and reliable (Figure S20, Supporting Information). The above experimental results demonstrate that LH@LA has excellent antitumor effects in animals.

### 2.7 Study of the Ability of LH@LA Improves the Tumor Immunosuppressive Microenvironment In Vivo

**Figure 7:**
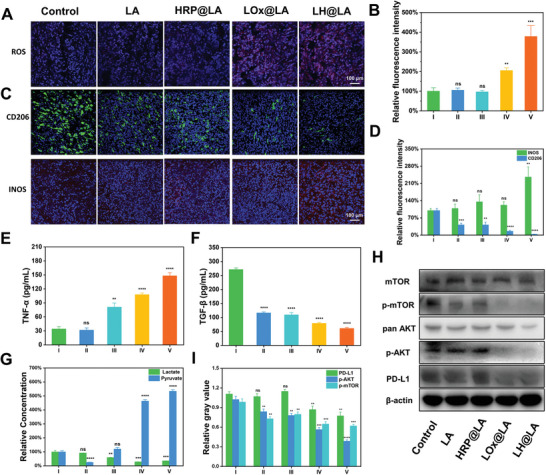
Study of the ability of LH@LA improve the immunosuppressive microenvironment of tumor tissues. A) ROS fluorescence imaging (Scale bar: 100 µm) and B) fluorescence quantification (*n* = 3) of tumor tissue sections after treatment in different administration groups. C) CD206, INOS fluorescence imaging (Scale bar: 100 µm), and D) fluorescence quantification (*n* = 3) of tumor tissue sections after treatment in different administration groups. Elisa kits were used to detect the concentrations of E) TNF‐α and F) TGF‐β in tumor tissues treated with different administration groups (*n* = 3). G) The kit detects the relative concentrations of lactate and pyruvate in tumor tissues treated with different administration groups (*n* = 3). H) Western Blots and I) Image software grey quantification (*n* = 3) is used to detect the expression of PD‐L1 protein as well as AKT‐mTOR pathway‐related proteins in tumor tissues treated with different administration groups. (I: Control, II: LA, III: HRP@LA, IV: LOx@LA, V: LH@LA). Statistical significance was calculated by t‐test for comparison between the two groups. ns: no significant, ^*^ *p* ≤ 0.05, ^**^ *p* ≤ 0.01, ^***^ *p* ≤ 0.001, ^****^ *p* ≤ 0.0001.

First, the ROS in the tumor tissue sections from different administration groups were stained by Dihydroethidium (DHE) and observed in CLSM. Similar to the results of the cellular experiments, LH@LA also produced a large amount of ROS in the tumor tissues, which played a pivotal role in its antitumor effect at the animal level. In contrast, no significant ROS production was observed in the tumor sections of the control group (**Figure** **7**A). Similarly, the fluorescence quantification results yielded consistent conclusions (Figure 7B). In addition, the tumor tissues with immunofluorescent staining for CD206 (M2 macrophage marker), INOS (M1 macrophage marker), CD 4, and CD 8. Compared to the other administration groups, the expression level of CD206 was significantly decreased and the expression level of INOS was significantly increased in the tumor tissues of the LH@LA group (Figure 7C). Similarly, the results obtained by fluorescence quantification are consistent with the experimental phenomena observed by fluorescence imaging described above (Figure 7D). In addition, a similar method was used to characterize the number of CD 4^+^ and CD 8^+^ T cells in tumor tissues from different administration groups. As shown in Figure S21 (Supporting Information), there was a significant increase in the number of CD 4^+^ and CD 8^+^ T cells in the LH@LA group.

In addition, the pro‐inflammatory factor TNF‐α and anti‐inflammatory factor transforming growth factor‐β (TGF‐β) in tumor tissues were quantified by Elisa assay kit. As shown in Figure 7E,F, as speculated is that the inflammatory factor TNF‐α concentration was significantly elevated in the LH@LA group compared to the other remaining groups, while the anti‐inflammatory factor TGF‐β concentration was significantly lower than in the remaining groups. Furthermore, D/L‐lactate and pyruvate concentrations in tumor tissues were measured by D/L‐lactate assay kit and pyruvate assay kit. As expected, the LH@LA group's D/L‐lactate concentration in tumor tissues decreased to one‐third of the control group, while pyruvate concentration was elevated to more than five folds of the control group (Figure 7G). The concentration of D‐lactate in tumor tissues was significantly increased at different time points, which is crucial for the improvement of the tumor microenvironment (Figure S22, Supporting Information). More importantly, the decrease of L‐lactate concentration is meaningful for down‐regulating the expression of PD‐L1 and inhibiting the AKT‐mTOR metabolic pathway in tumor cells. Therefore, western blot assays were used to examine the expression of PD‐L1, AKT, mTOR, p‐AKT, and p‐mTOR in tumor tissues. As shown in Figure 7H, the expression of PD‐L1, p‐AKT, and p‐mTOR in the LOx@LA group as well as in the LH@LA group was significantly lower than that in the remaining groups. In addition, similar conclusions were reached by quantitative analyses of protein blots (Figure 7I). The above experimental results demonstrate that LH@LA still has good antitumor activity in mice through LH@LA metabolism and catalysis by the LOx/HRP cascade enzyme system.

Currently, probiotic‐based cancer treatment strategies have shown promising antitumor efficacy.^[^ ^18^ ^]^ However, their clinical translation still faces many challenges, especially safety, quality control, and dosing regimens. It is worth noting that some of these probiotic‐mediated cancer treatments have entered the clinical trial stage, which shows that probiotic‐based systems still have great clinical application prospects.^[^ ^19^ ^]^ To reduce the systemic side effects caused by the probiotic carrier, we chose a safer and more efficient local injection method to improve efficacy and minimize side effects. At the same time, to more comprehensively evaluate the biocompatibility of LH@LA intratumoral delivery, we also measured the levels of reactive oxygen species (ROS) in major organs and endotoxin levels in serum to assess the systemic toxicity of the treatment. For this purpose, we used dihydroethidium (DHE) staining to detect ROS in major organs and an endotoxin detection kit to measure endotoxin levels in serum. As shown in Figures S23 and S24 (Supporting Information), there was no significant ROS production in the major organs of mice treated with LH@LA, and the endotoxin levels in the serum of these mice did not show any significant increase. These results indicate that LH@LA exhibits excellent biocompatibility. The above results once again demonstrate that the cancer treatment strategy through local injection of LH@LA has good biosafety and does not cause systemic toxic side effects in mice, which provides strong support for the future clinical transformation of engineered probiotics.

## 3 Conclusion

In summary, we have developed an engineered probiotic (LH@LA) armed with a polymeric stealth coating co‐loaded with LOx and HRP by an original in situ polymerization method. The polymer coating can efficiently load a variety of enzymes while maintaining bacterial activity and can enable it to escape the phagocytosis of macrophages, thereby effectively delivering drugs deep into the tumor. The LOx/HRP enzyme cascade reaction system can effectively reduce the concentration of overexpressed L‐lactate and GSH in tumor cells while inhibiting the oxidative phosphorylation process in the tumor, reducing the energy supply of tumor cells, and destroying the antioxidant defense system inside tumor cells. More importantly, through LA metabolism, it can compete with tumor cells for nutrients and produce D‐lactate with immunomodulatory effects, thereby effectively promoting the repolarization of M2 macrophages to M1 macrophages. Moreover, this engineered probiotic can consume lactate in tumor cells from the inside out, effectively reduce the expression of PD‐L1 and inhibit the activation of the AKT‐mTOR metabolic pathway, thereby synergizing lactate metabolism‐immunity‐enzyme dynamic therapy to effectively inhibit tumor development. This safe, efficient, simple, and easy‐to‐operate engineered probiotic design provides a new idea for building a functional live bacteria antitumor platform and has good prospects for clinical transformation.

## 4 Experimental Section

### Materials

*Lactobacillus acidophilus* (Moro) Hansen and Mosquot (LA) was purchased from BNCC, and Lactate Oxidase (LOx) was purchased from Anhui Senrise Technology Co., Ltd. 2‐(dimethylamino)ethyl methacrylate (2‐DM) Horseradish peroxidase (HRP) was purchased from Sigma–Aldrich. BCA (bicinchoninic acid) protein assay was purchased from Thermo. Phosphate buffered saline (PBS), bovine serum albumin (BSA) and JC‐1 (5′, 6, 6′‐tetrachloro‐1, 1′, 3, 3′‐tetraethylbenzimidazolylcarbocyanine iodide) was supplied by Solarbio Technology Co., Ltd., antifade mounting medium with DAPI (4′,6‐diamidino‐2‐phenylindole) and TUNEL (terminal transferase uridyl nick end labeling) apoptosis detection kit (Alexa Fluor 647, catalog number: 40308ES20) and DCFH‐DA (dichlorodihydrofluorescein diacetate) were obtained from Yeasen Biotechnology Co. Ltd. Akt (pan) (C67E7) Rabbit mAb #4691 and mTOR (7C10) Rabbit mAb #2983 were obtained from Cell Signaling Technology. Phospho‐AKT (Ser473) Recombinant antibody (catalog number: 80455‐1‐RR), Phospho‐mTOR (Ser2448) Monoclonal antibody (catalog number: 67778‐1‐Ig), iNOS antibody (CL647‐18985) and Arg‐1 antibody (CL594‐66129) were obtained from Proteintech. PD‐L1 Mouse Monoclonal Antibody‐ #BF8035 was obtained from Affinity Biosciences. Alkaline phosphatase (ALP) detection kits, aspartate aminotransferase (AST) detection kits, alanine aminotransferase (ALT) detection kits, albumin (ALB) detection kits, ATP content detection kits, DL lactate detection kits and D lactate detection kit were obtained from Nanjing Jiancheng Technology Co.Ltd. Glucose detection kit were obtained from Beyotime Biotechnology. NAD^+^/NADH colorimetric Assay kits were obtained from Elabscience. Mouse Endotoxin (ET) ELISA Kit was supplied by Shanghai Zcibio Technology Co. Ltd.

### Preparation of LH@LA

The synthesis of LH@LA was by a simple one‐step in situ polymerization method. LA (5 mL, OD = 0.2) suspension was added with 60 µL 2‐(dimethylamino) ethyl methacrylate and stirred at 500 rpm for 30 min. Then 1 mg LOx and 1 mg HRP were added and stirred at 500 rpm for another 30 min. Finally, added 60 mg acrylmide and 35 mg N, N‐methylene bis‐acrylamide under nitrogen protection and stirred at 500 rpm for 2. Then added 48 µL TEMED and 24 mg ammonium persulfate (APS) and stirred for a further 2. And LH@LA was then obtained by several centrifuges and suspension.

### Characterization of LH@LA

The surface charge of LH@LA was quantified by Laser Particle Size and Zeta Potential Analyzer (Malvern Instruments, ZS 90). Meanwhile, the particle morphology was observed by Scanning Electron Microscopy (SEM) (ZEISS, Germany).

### Detection of Free Radicals In Vitro

To investigate the efficiency of LOx@LA in producing ROS in vitro, the production of free radicals was verified by TMB color reaction. Briefly, 10 µL (OD = 0.2) of LOx@LA was added to 100 µL of L‐lactate solution (10 mm) and the reaction was performed for 10 min at 37 °C, and the supernatant was collected by centrifugation. Subsequently, 2 µL of HRP (0.06 mg mL^−1^) and 100 µL of chromogenic solution (250 µL of TMB added to 1 mL of PBS) were added, and it was combined for 15 min before measuring the absorption spectrum between 520 and 750 nm.

### Cell Culture

4T1 (Mouse Breast Carcinoma Cells) and RAW 264.7 (Mouse mononuclear macrophage leukemia cells) were cultured in RPMI 1640 cell culture media, with 10% fetal bovine serum, penicillin (100 U mL^−1^), and streptomycin (100 µg mL^−1^). And cells were kept in an ESCO cell culture incubator with 37 °C and 5% CO_2_.

### Cell Viability Assay

The cell viability assay was mainly verified by Calcein‐AM /PI Double Stain Kit. In brief, 4 × 10^5^ 4T1 cells were uniformly spread in 12‐well cell culture plates and cultured overnight. After the cells grew to a certain density, the corresponding group of materials of 5 µL (OD = 0.2) was added, and after 14 h, the cells were stained by the kit and observed in the confocal laser scanning microscope (CLSM).

### Apoptosis Assay

4T1 cells (4 × 10^5^ cells) were inoculated on 12‐well cell culture plates and treated with materials (5 µL, OD = 0.2) from each group for 14 h. Apoptosis was evaluated by membrane connexin V‐FITC staining and PI labeling. The procedure was as follows: the collected cells were washed with cold PBS and re‐suspended in 0.5 mL PBS buffer. Then 10 µL propyl iodide (PI) and 5 µL fluorescein isothiocyanate (FITC) coupled membrane coupled protein V were stained at 37 °C for 15 min without light. Data were collected and analyzed by flow cytometry. The fluorescence test of apoptosis was similar to the above.

### Uptake of Protein by 4T1 Cells

In order to verify whether the LOx/HRP loaded on the surface of LA can fall off and play a role in tumor cells. First, FITC‐BSA@LA was synthesized, then it was added to a pre‐laid cell crawl sheet containing cells. Subsequently, the state of the FITC‐BSA at different time points and the FITC‐BSA entering the cell were observed by confocal laser confocal microscopy at different time points.

### Changes of Intracellular ROS Induced by LH@LA

Intracellular ROS levels were measured by DCFH‐DA. The increase in fluorescence intensity indicated the increase in ROS level. In brief, 1 × 10^6^ 4T1 cells were inoculated on six‐well cell culture plates and treated with PBS, LA, LOx@LA, HRP@LA, LH@LA (OD = 0.2, 10 µL) separately or 4T1 cells were cultured at a density of 5 × 10^4^ cells per well on the cell crawl sheet in 48‐well plates and treated with PBS, LA, LOX@LA, HRP@LA, LH@LA (OD = 0.2, 1.25 µL) separately. After 8 h, the culture medium was replaced with DCFH‐DA stock solution (10 nm) and incubated for 30 min. Reactive oxygen species were detected by flow cytometry and CLSM (Zeiss LSM5, Germany).

### Repolarization of RAW 264.7 Macrophage Induced by LH@LA

The polarization of RAW 264.7 macrophages was verified by flow cytometry and immunofluorescence. In brief, 1 × 10^6^ 4T1 cells were spread into six‐well plates. When the cell density reached 50%, the culture medium was changed into a serum‐free medium. 25 ng mL^−1^ IL‐4 and 10 µL (OD = 0.2) LA, HRP@LA, LOx@LA, LH@LA was added in each group and treated for 8 h. At the same time, a blank control group (culture without serum medium) was set up. The cells were then collected and cleaned with phosphate buffer (PBS). Finally, CD 11b (macrophage surface marker) and CD 86 (M1 macrophage surface marker) on the cell surface were stained for 30 min. Data were collected and analyzed by flow cytometry. Similarly, INOS (M1 macrophage marker) and ARG‐1 (M2 macrophage marker) on the surface of RAW 264.7 macrophages were stained by immunofluorescence. In brief, 5 × 10^4^ cells were placed on a 48‐well plate pre‐laid with a cell crawl sheet and cultured overnight. Then each group of materials was added and treated for 8 h. The cells were then fixed, transmembrane, and stained. And observed by CLSM.

### Intracellular Mitochondrial Membrane Potential (MMP Assay (JC‐1))

MMP was detected by observing fluorescence shifts using the mitochondrial permeability fluorescent dye JC‐1. Mitochondrial activity was assessed with fluorescence in green (JC‐1 monomer) and red (JC‐1 aggregates) using 535 and 595 nm emission filters, respectively. In brief, 5 × 10^4^ cells were placed on a 48‐well plate pre‐laid with a cell crawl sheet and cultured overnight. After treated by each group material for 8 h and washed twice with PBS, the cells were incubated with JC‐1 (5 µg mL^−1^) at 37 °C and 5% CO_2_ for 20 min. And fluorescence changes were observed by CLSM.

### Western Blotting

For PD‐L1, pan‐AKT, and phospho‐AKT, 15 µg of protein was separated by 12.5% SDS‐gel electrophoresis and transferred to a PVDF membrane. The membranes were closed with a closure solution for 2 h at room temperature. After washing the membranes with 1 × TBST (Tris‐buffered saline with tween), the blots were incubated overnight at 4 °C with the corresponding primary antibodies against PD‐L1, AKT (pan), Phospho‐AKT and β‐actin. Thereafter, the membranes were washed with 1× TBST and incubated with the corresponding secondary antibodies. Finally, visualization was performed with ECL (enhanced chemiluminescence) detection reagent. For mTOR and phospho‐mTOR, only 12.5% SDS‐gel electrophoresis was changed to 7.5% SDS‐gel electrophoresis, all other conditions remain the same.

### Study on the In Vivo Distribution of LH@LA

The in vivo distribution of LH@LA was analyzed by collecting major organs, brain, tumor, and blood from mice at different time points after injection. Specifically, equal masses of these tissues and blood samples were collected at various time points post‐injection and cultured in MRS medium for 24 h. After culturing, the samples underwent multiple rounds of centrifugation, washing, and resuspension in PBS. The OD_600_ values were then measured, and these values were converted to bacterial counts.

### In Vivo Antitumor Studies

Female BALB/c mice weighing 16–20 g were fed at the Experimental Animal Center of Xiamen University. Animal care guidelines of Xiamen University were used throughout the animal experiments. The experiments were approved by the Xiamen University Laboratory Animal Management Regulations (Animal experiment ethical review number: XMULAC20230118). To form solid tumors in mice, 100 µL 4T1 cell suspension (2 × 10^6^ cells) was injected subcutaneously. When the tumor volume reached ≈80–120 mm^3^, 100 µL (OD = 0.2) of various drug preparations (PBS, LA, HRP@LA, LOx@LA, LH@LA) were injected intratumorally every two days. The body weight and tumor size of the mice were monitored throughout the treatment. After 14 days of drug treatment, whole blood was collected for serum analysis. Mice were executed and tumors were excised for other assessments. Tumor volume was calculated using the following equation:

(1)

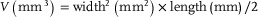




### H&E (Hematoxiline‐Eosin) Staining

The collected tumors and other major organs were successively fixed with 4% paraformaldehyde fix solution for 24 h, respectively. Tumors and organs were embedded in OCT compound and cut into 6–7 µm thick frozen sections for the following biochemical analyses. The sections were stained with hematoxylin and eosin and visualized by light microscopy (Olympus BX43 biomicroscope).

### Tissue Immunofluorescence Staining

The expression of related proteins in tumor tissues was verified by immunofluorescence staining. In brief, tumors were cut into 6–7 µm thick frozen sections. Subsequently, the related proteins were characterized by immunofluorescence staining methods similar to those described above. Finally, it was observed in CLSM.

### Tumor Tissue and Major Organs ROS Staining

First, fresh tumor tissues and major organs were prepared as frozen sections. After sectioning, the fresh tumor tissues and major organs were stained with DHE for 30 min. Then, the sections were mounted with an antifade mounting medium with DAPI to seal the tissues. Finally, fluorescent imaging was performed using a confocal laser scanning microscope (CLSM).

### D‐Lactate Concentration Measurement in Tumor Tissue

The concentration of D‐lactate in tumor tissues was determined using a D‐lactate concentration assay kit. Briefly, equal masses of tumor tissue were taken at different time points post‐administration, and the tumor tissues were homogenized and centrifuged. The supernatant was collected. Subsequently, the D‐lactate concentration in the supernatant was measured using the assay kit.

### Blood Endotoxin Level Testing

The detection of endotoxins in serum was performed using a Mouse ETE ELISA Kit. In brief, the kit was equilibrated to room temperature for 30 min. Standard wells and sample wells were set up; 50 µL of different concentrations of standards were added to the standard wells, while 50 µL of the test samples were added to the sample wells; no additions were made to the blank wells. To both standard and sample wells, 100 µL of horseradish peroxidase (HRP)‐conjugated detection antibody was added, and the reaction wells were sealed with a plate sealer. The plate was then incubated in a water bath or incubator at 37 °C for 60 min. After incubation, the liquid was discarded and the wells were dried on absorbent paper. Each well was filled with 350 µL of wash buffer, left to stand for 1 min, and then the wash buffer was discarded and the wells dried on absorbent paper. This washing process was repeated five times. Finally, a color development solution was added, followed by a stop solution, and the absorbance was measured at 450 nm. A standard curve was plotted, and the data were processed accordingly.

### Blood Biochemical Examination

To assess the toxicity of the materials to the liver and kidneys, serum concentrations of alkaline phosphatase (ALP), aspartate aminotransferase (AST), alanine aminotransferase (ALT), and albumin (ALB) were measured in mice in each dosing group with reference to the manufacturer's protocol.

### Statistical Analysis

Values were shown as mean ± standard deviation, a GraphPad Prism 8.0.2 was applied for statistical analysis. Unpaired *t*‐tests were used for statistical analysis to compare statistical significance. ns: *p* ≥ 0.05, ^*^ *p* < 0.05, ^**^ *p* < 0.01 and ^***^ *p* < 0.001, ^****^ *p* < 0.0001 were respectively recognized as statistically significant, highly significant and very significant.

## Conflict of Interest

The authors declare no conflict of interest.

## Supporting information



Supporting Information

## Data Availability

The data that support the findings of this study are available on request from the corresponding author. The data are not publicly available due to privacy or ethical restrictions.
