# Supplementary material for: Natural Enzyme‐Loaded Polymeric Stealth Coating‐Armed Engineered Probiotics by Disrupting Tumor Lactate Homeostasis to Synergistic Metabolism‐Immuno‐Enzyme Dynamic Therapy
Source: Adv Sci (Weinh). 2025 Feb 28;12(16):2417172. doi: 10.1002/advs.202417172 (PMC12021032; doi:10.1002/advs.202417172)
Supplement: Supplementary file 1 — Supporting Information [file ADVS-12-2417172-s001.docx]

**Supporting Information**

for

**Natural Enzyme-Loaded Polymeric Stealth Coating-Armed Engineered Probiotics by Disrupting Tumor Lactate Homeostasis to Synergistic Metabolism-Immuno-Enzyme Dynamic Therapy**

*Liuzhou, Mao^[a]^, Bahriman, Xarpidin^[a]^, Rui, Shi^[a]^, Yuting, Lin^[a]^, Haohua, Hu^[a]^, Caisheng, Wu^* [a]^, Zheng, Luo^*[a]^, Yun-Long Wu^*[a]^*

[a] L. Mao, B. Xarpidin, R. Shi, Y. Lin, H. Hu, C. Wu, Z. Luo, Y.-L. Wu, State Key Laboratory of Cellular Stress Biology, Fujian Provincial Key Laboratory of Innovative Drug Target Research, School of Pharmaceutical Sciences, Xiamen University, Xiamen 361102, China.

E-mail: wucsh@xmu.edu.cn; zhengluo@stu.xmu.edu.cn; wuyl@xmu.edu.cn


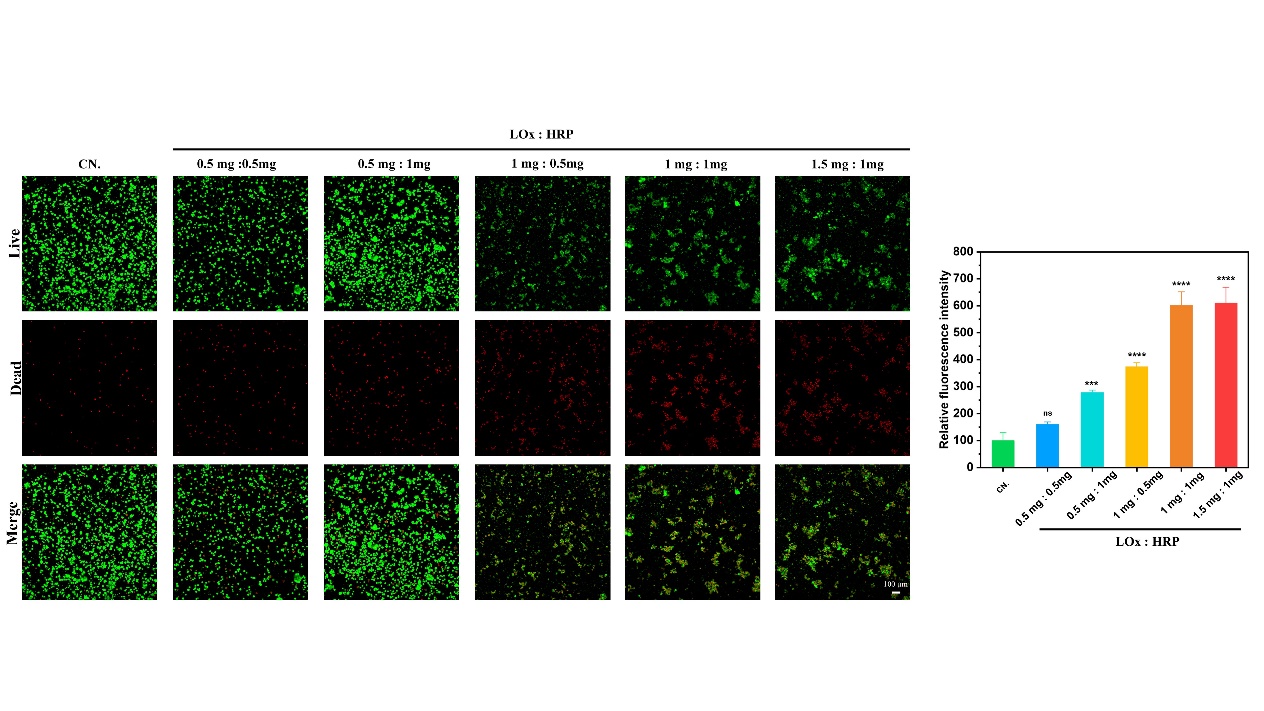


**Figure S1.** Live/dead staining and relative fluorescence intensity quantification of 4T1 cells after treatment with different ratios of LOx and HRP mixture (red fluorescence indicating dead cells). (Scale bar: 100 μm)


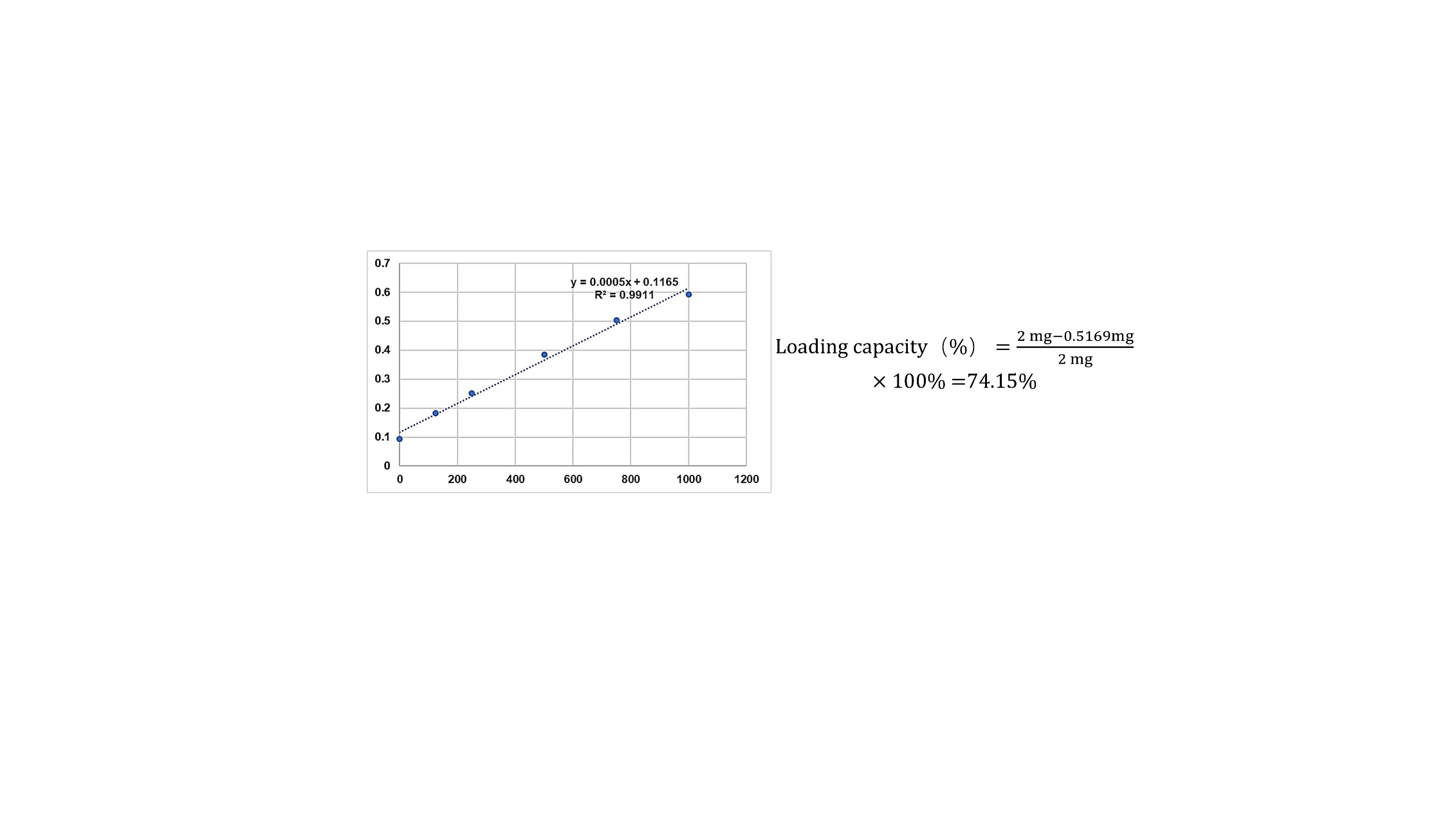


**Figure S2.** Detection of enzyme loading after the reaction synthesis when 1 mg of LOx and 1 mg of HRP are each added.

**
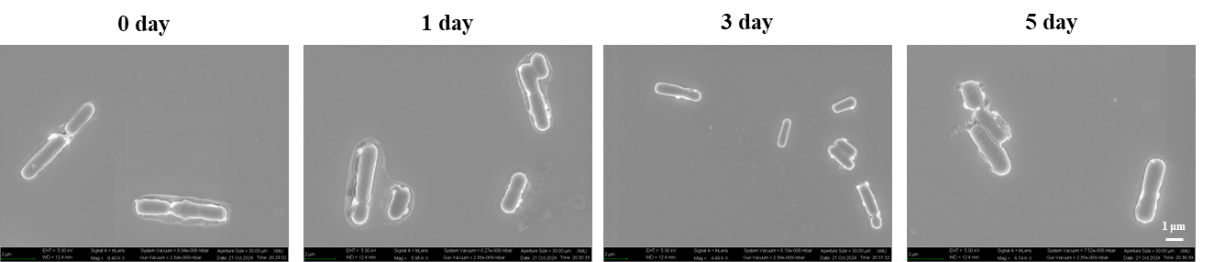
**

**Figure S3.** SEM images of LH@LA at different time points after synthesis (Scale bar: 1 μm).


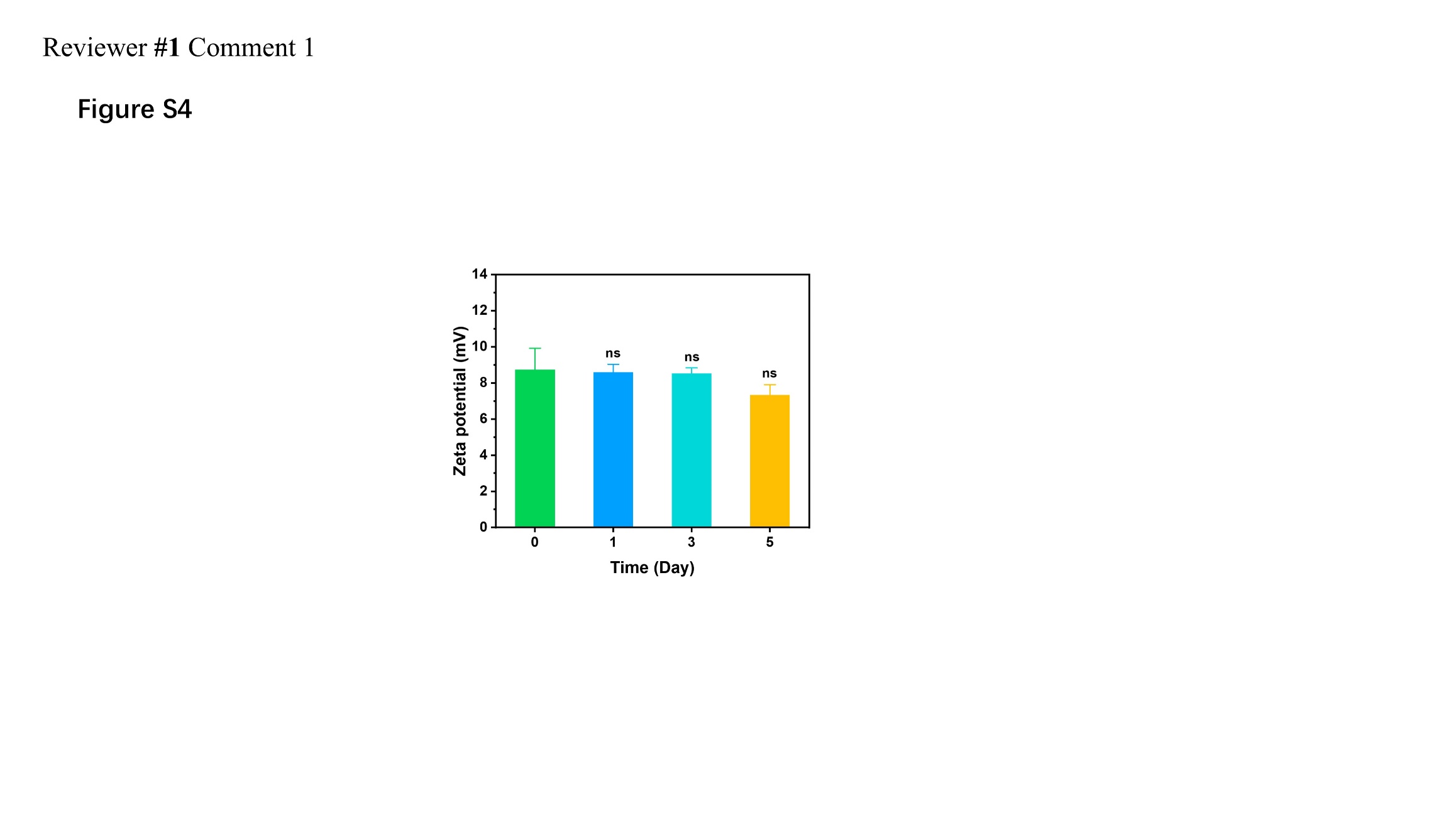


**Figure S4.** Potential values of LH@LA at different time points after synthesis.

**
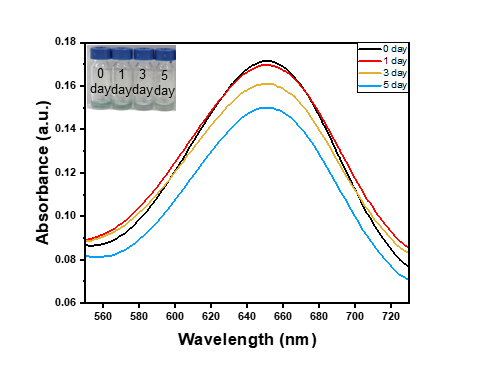
**

**Figure S5.** TMB colorimetric reaction to verify the catalytic production of reactive oxygen species (ROS) from lactate by LOx@LA at different time points after synthesis.

**
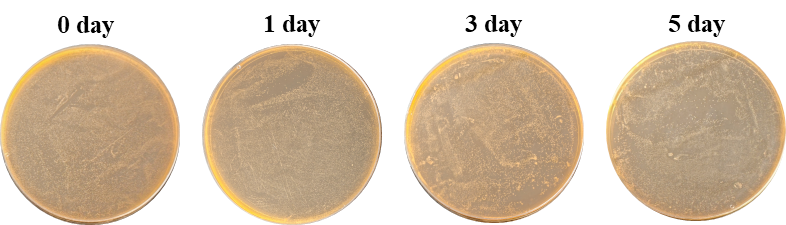
**

**Figure S6.** Cultivation images of LH@LA on MRS solid medium at different time points after synthesis.

**
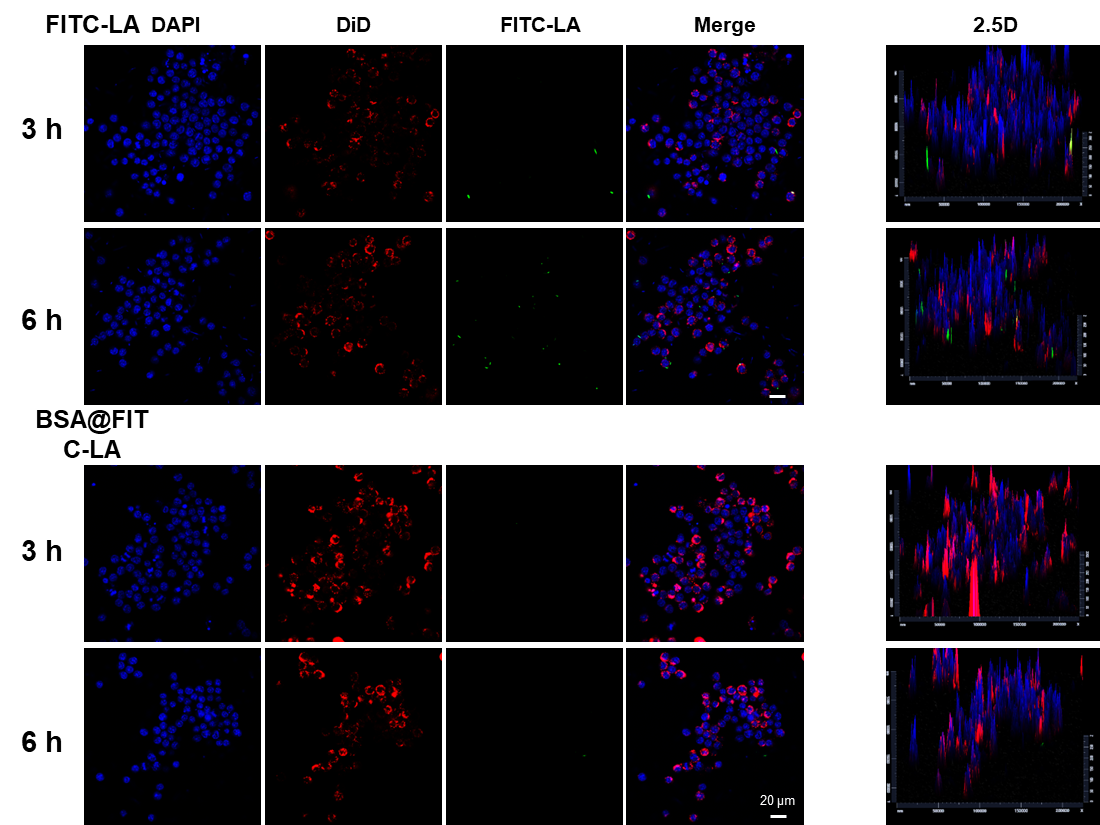
**

**Figure S7.** Fluorescence imaging of RAW 264.7 cell uptake of FITC-LA and BSA@FITC-LA (Scale bar: 20 μm).

**
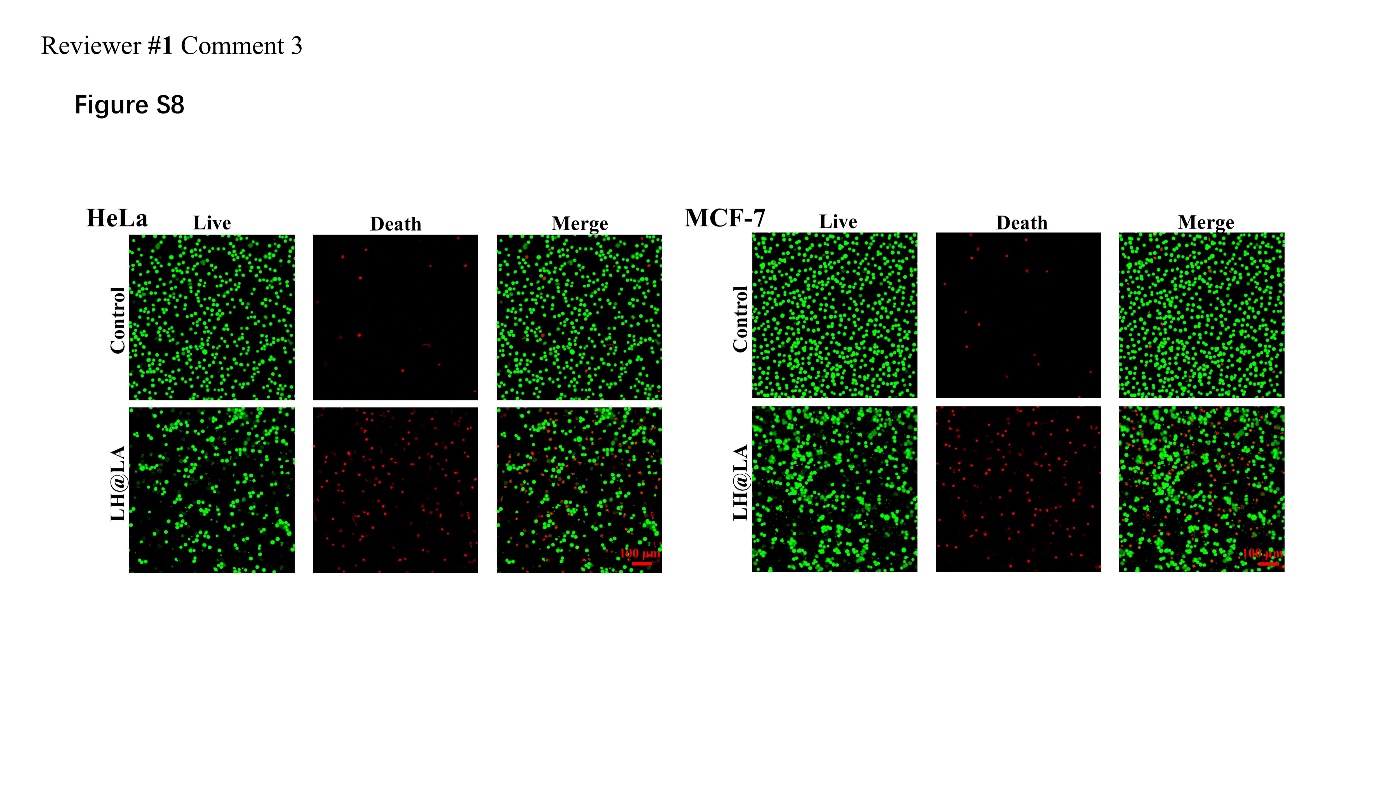
**

**Figure S8.** Fluorescence images of Live/Dead staining of HeLa and MCF-7 cells after treatment with LH@LA (Scale bar: 100 μm).

**
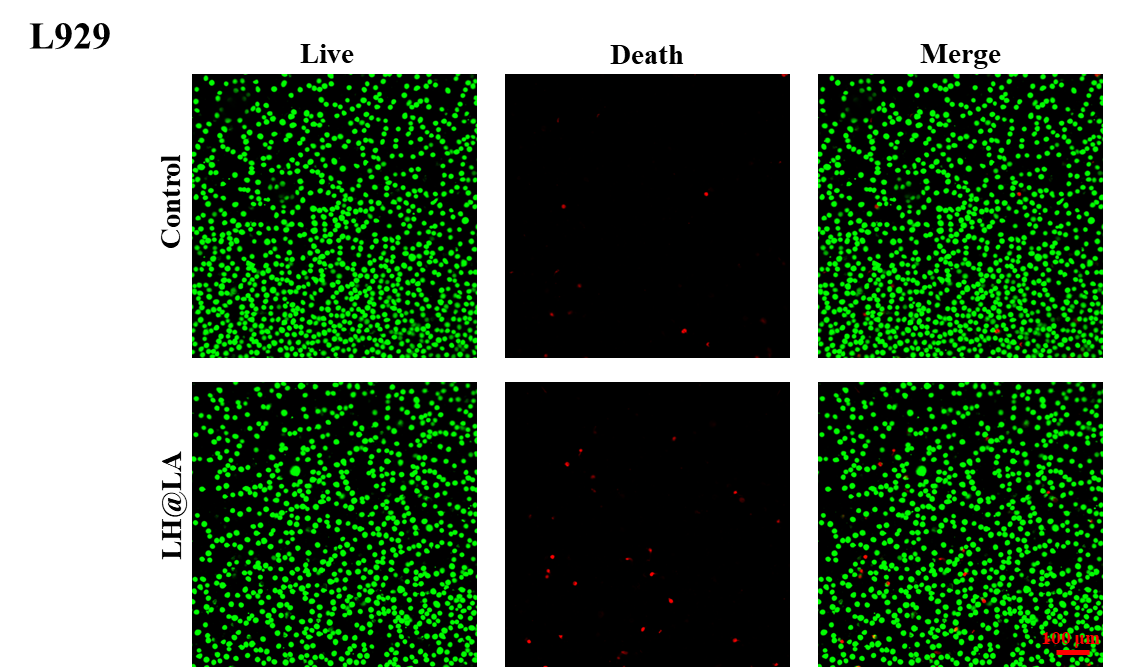
**

**Figure S9.** Fluorescence images of Live/Dead staining of L929 cells after treatment with LH@LA (Scale bar: 100 μm).


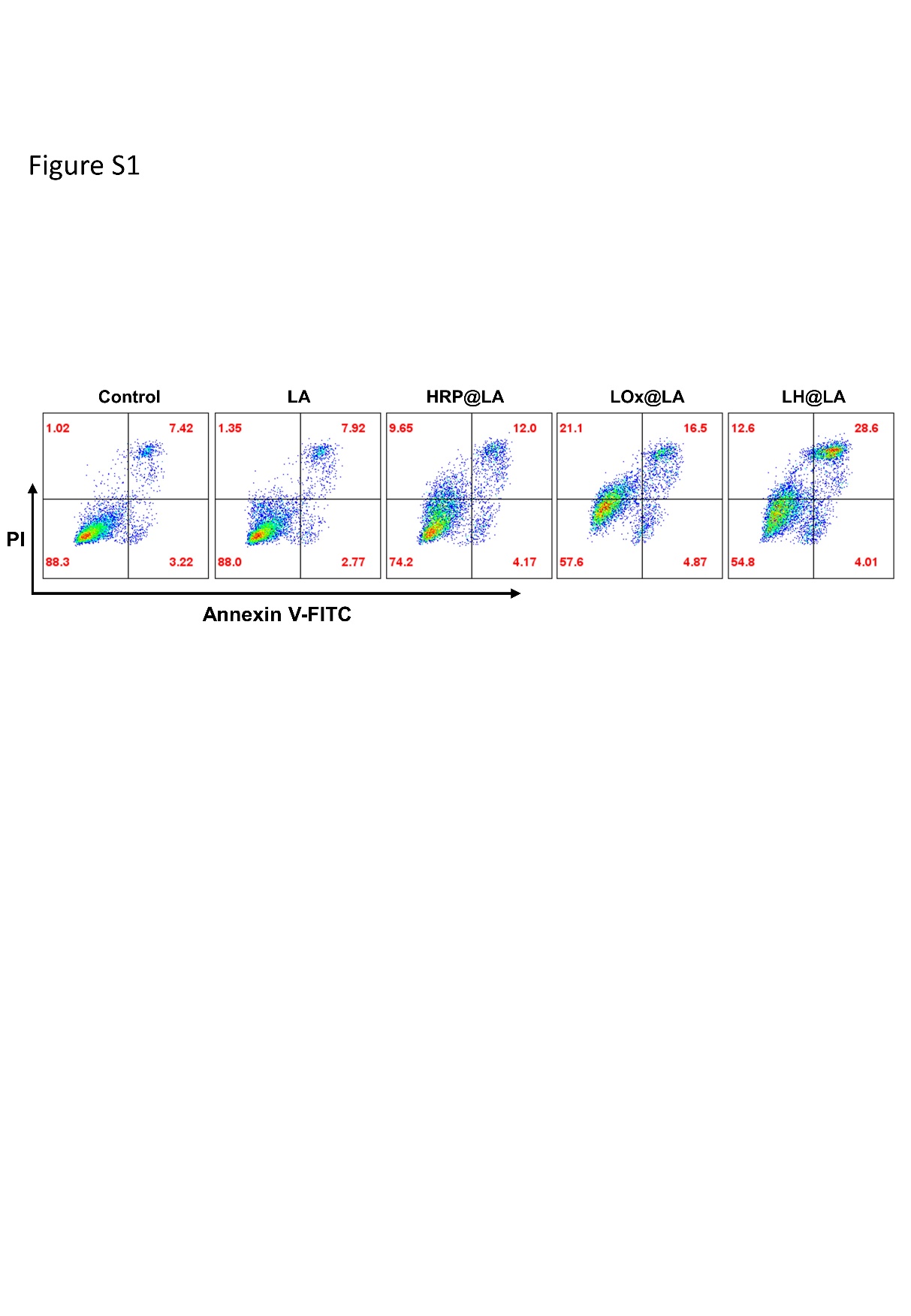


**Figure S10.** Flow cytometry detection of apoptotic cycles in 4T1 cells after treatment with different materials.


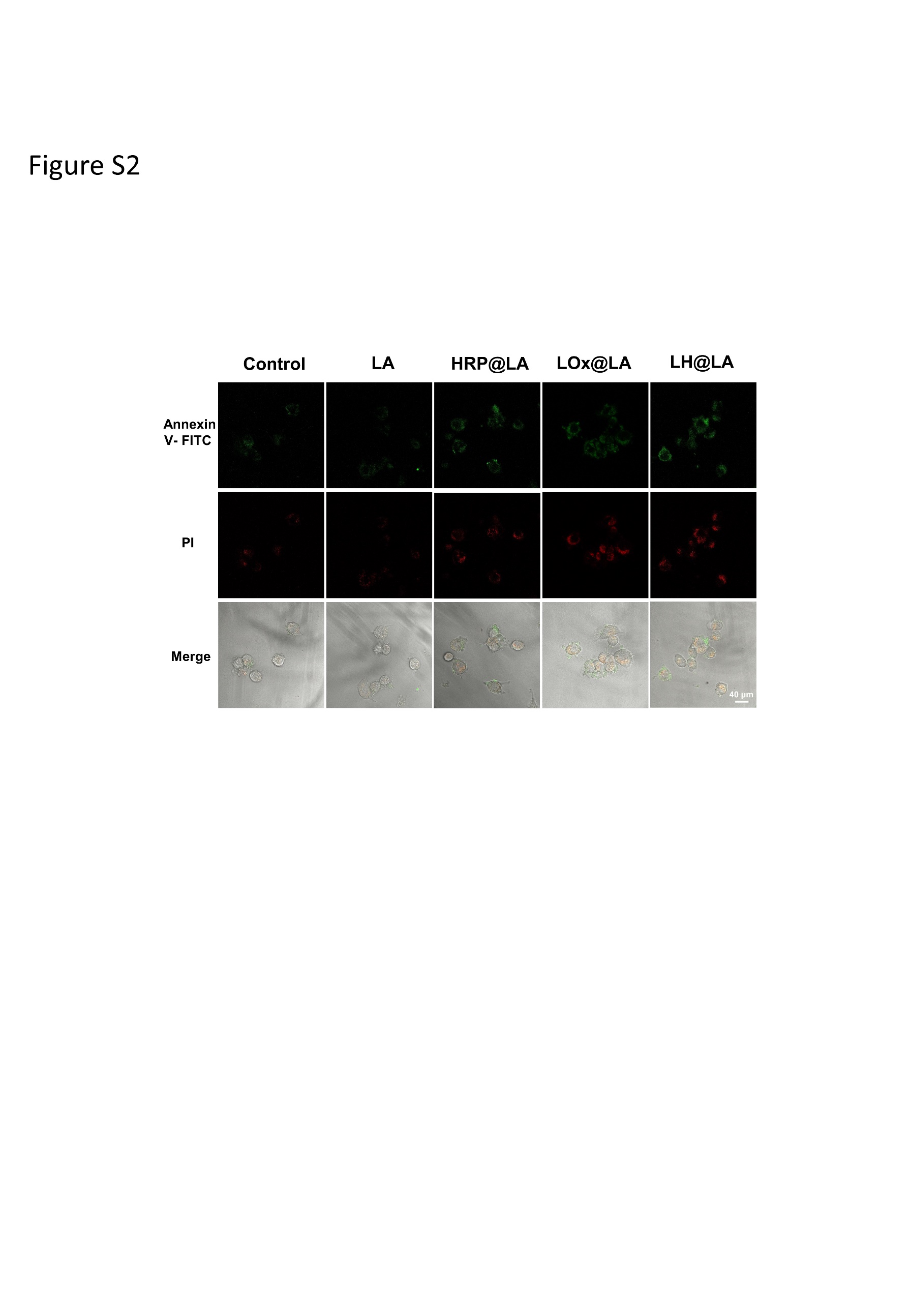


**Figure S11.** Apoptosis detection kit to detect CLSM fluorescence imaging of 4T1 cells after treatment with different materials (Scale bar: 40 μm).

**
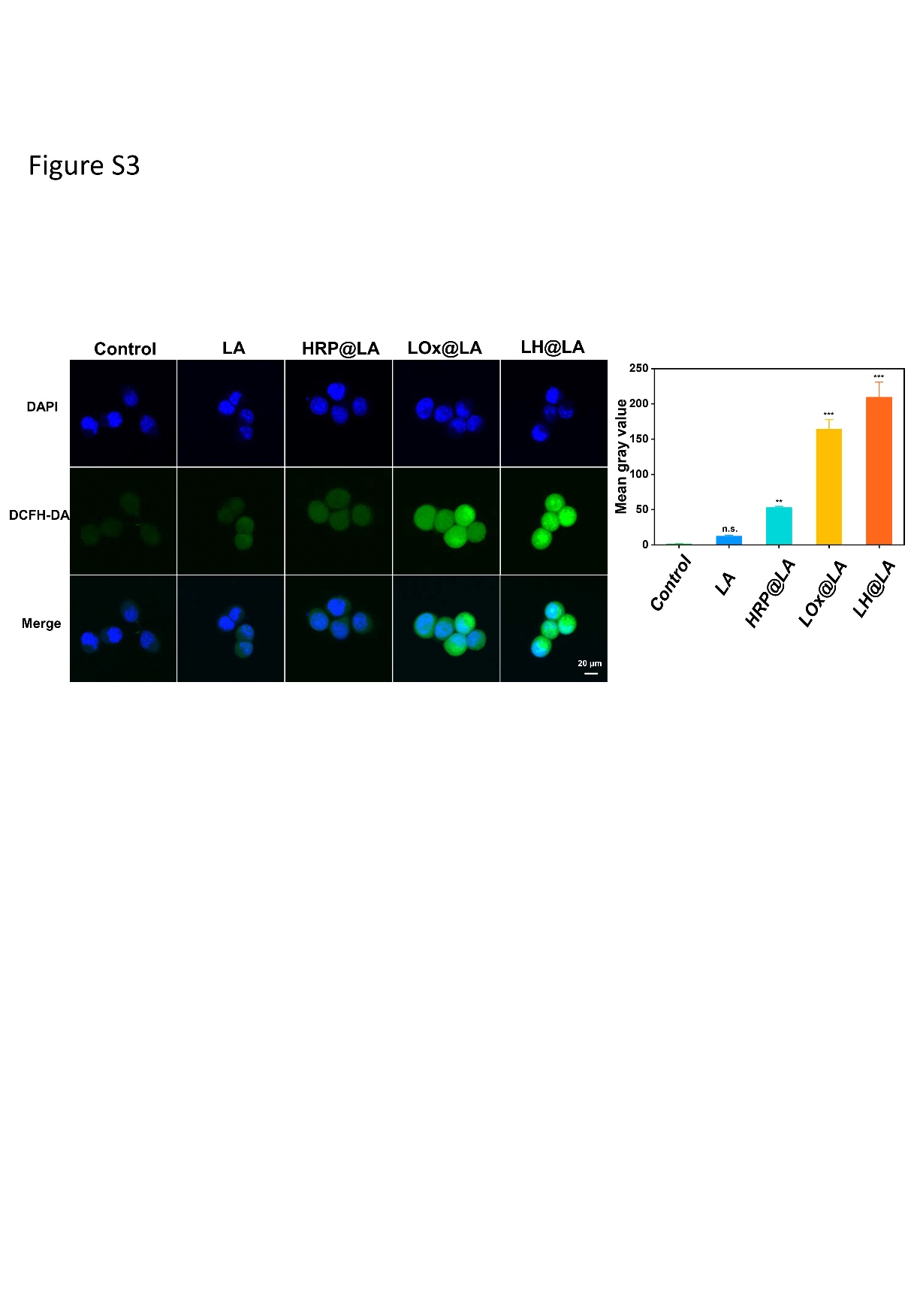
Figure S12.** Intracellular ROS fluorescence imaging (left image) (Scale bar: 20 μm) and fluorescence quantification (right bar chart).

**
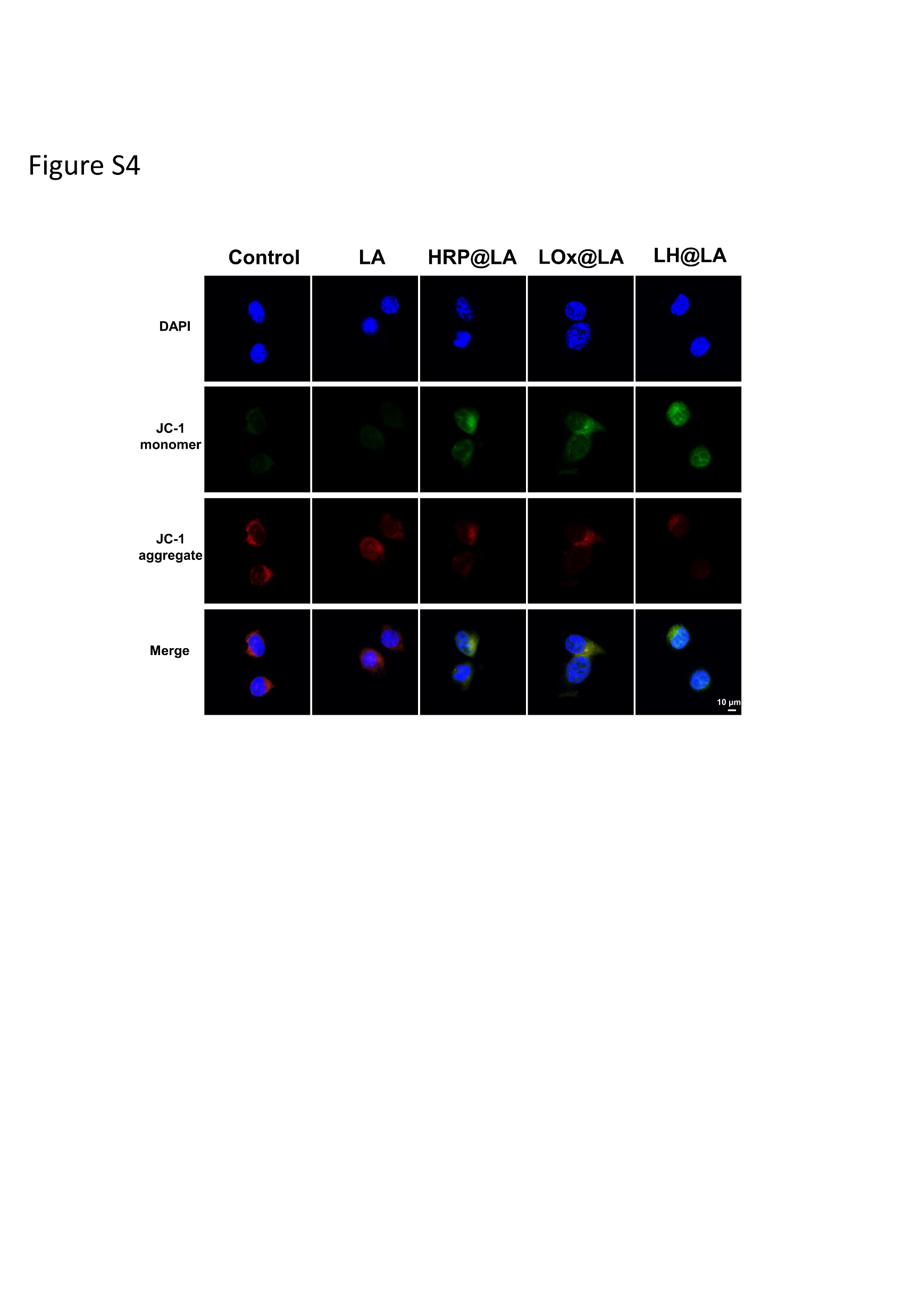
**

**Figure S13.** JC-1 detection of CLSM fluorescence imaging of mitochondrial membrane potential after treatment of 4T1 cells with different materials (Scale: 10 μm).


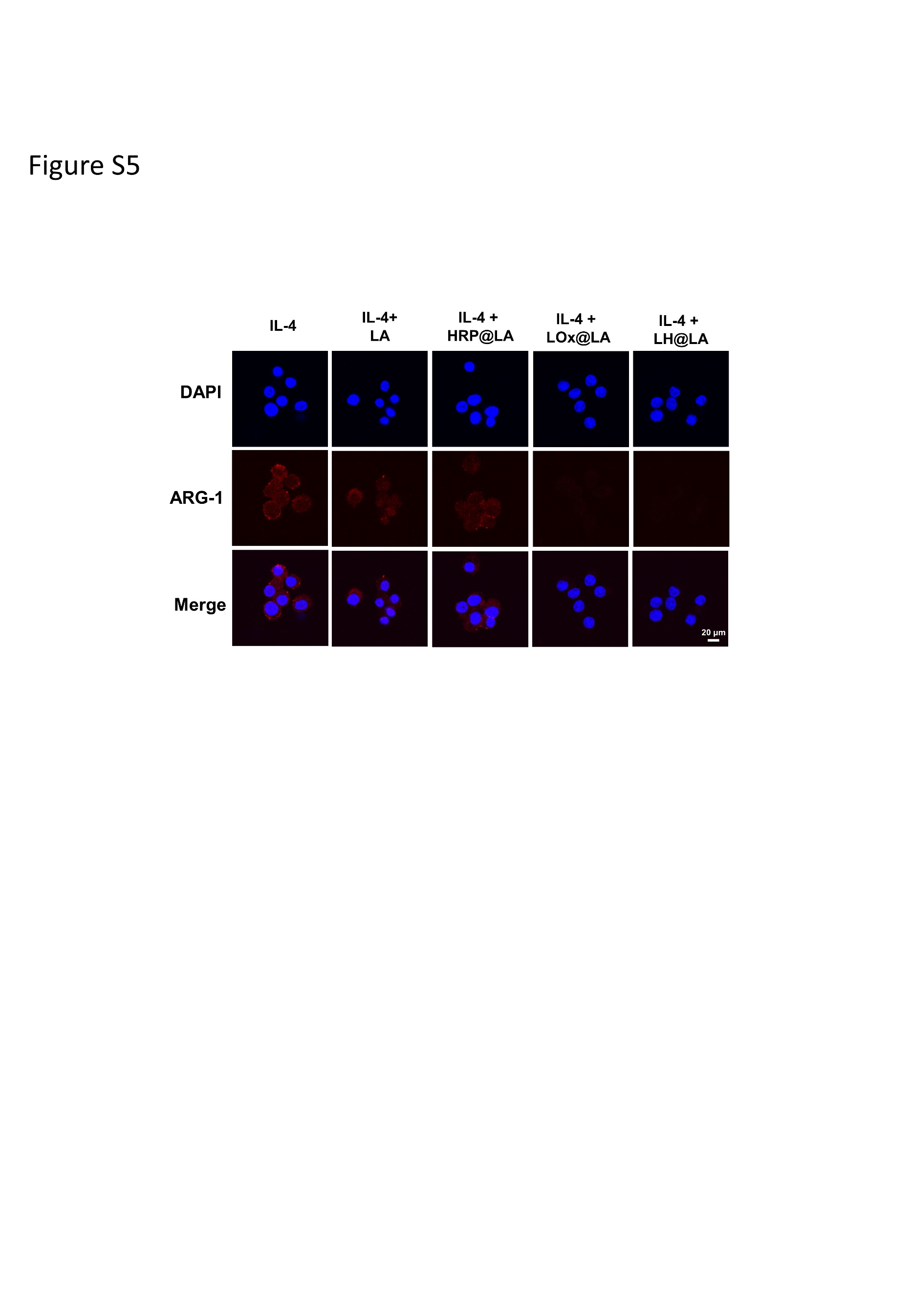


**Figure S14.** ARG-1 fluorescence imaging of RAW 264.7 macrophages treated with different administration methods (Scale bar: 20 μm).


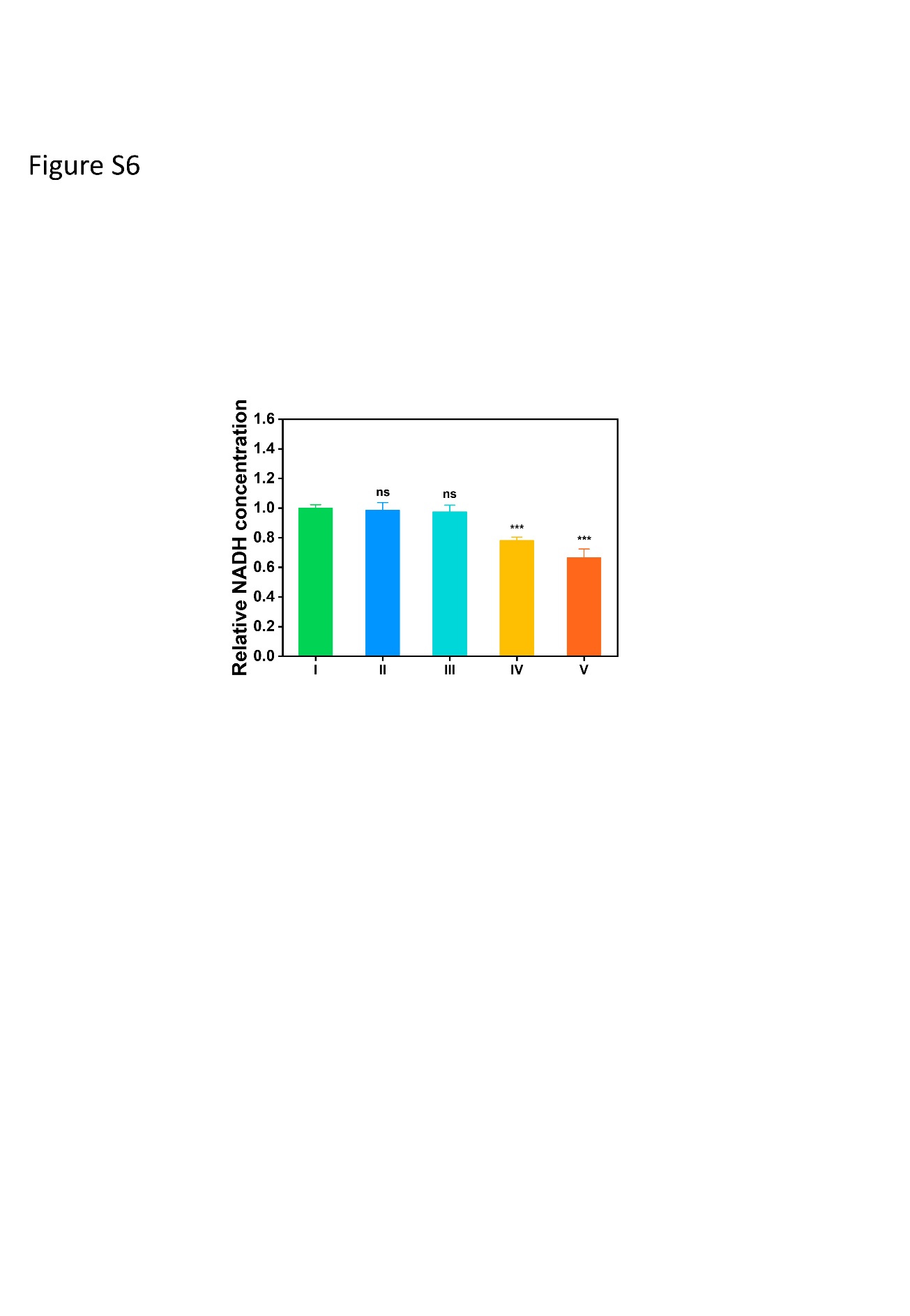


**Figure S15.** Relative intracellular NADH concentrations after treatment of 4T1 cells with different materials (n=3). (I: Control, II: LA, III: HRP@LA, IV: LOx@LA, V: LH@LA). Statistical significance was calculated by t-test for comparison between two groups. ns: no significant, *P ≤ 0.05, **P ≤ 0.01, ***P ≤ 0.001.


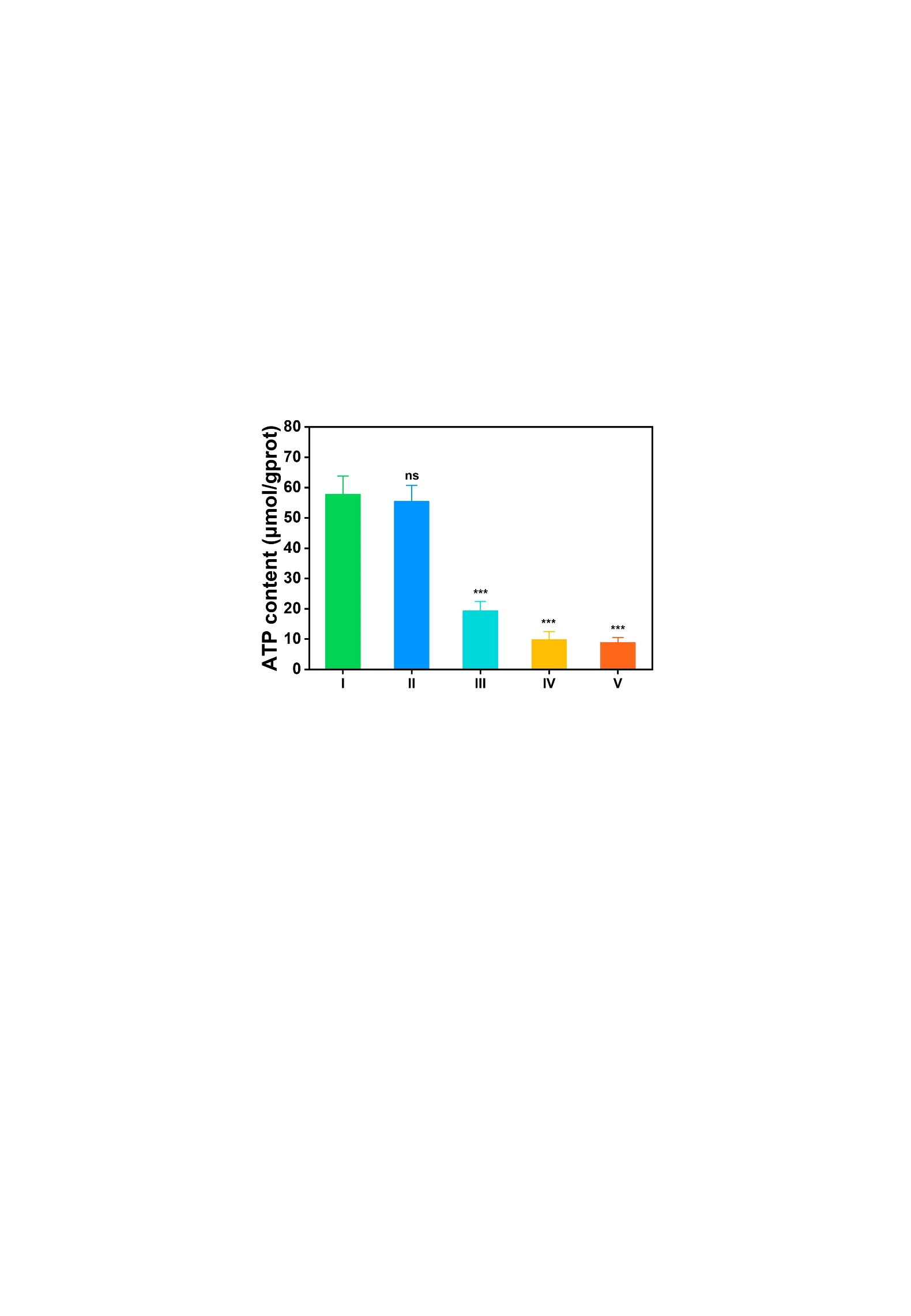


**Figure S16.** Intracellular ATP concentration after treatment of 4T1 cells with different materials. Statistical significance was calculated by t-test for comparison between two groups (n=3). (I: Control, II: LA, III: HRP@LA, IV: LOx@LA, V: LH@LA). ns: no significant, *P ≤ 0.05, **P ≤ 0.01, ***P ≤ 0.001.


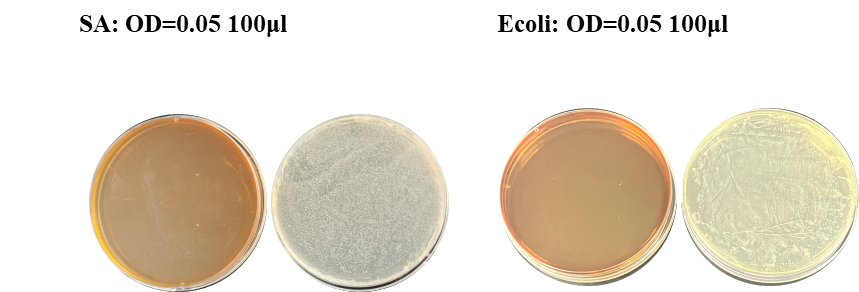


**Figure S17.** Cultivation images of Staphylococcus aureus (SA) and Escherichia coli (E. coli) on MRS solid medium (left) and LB solid medium (right).


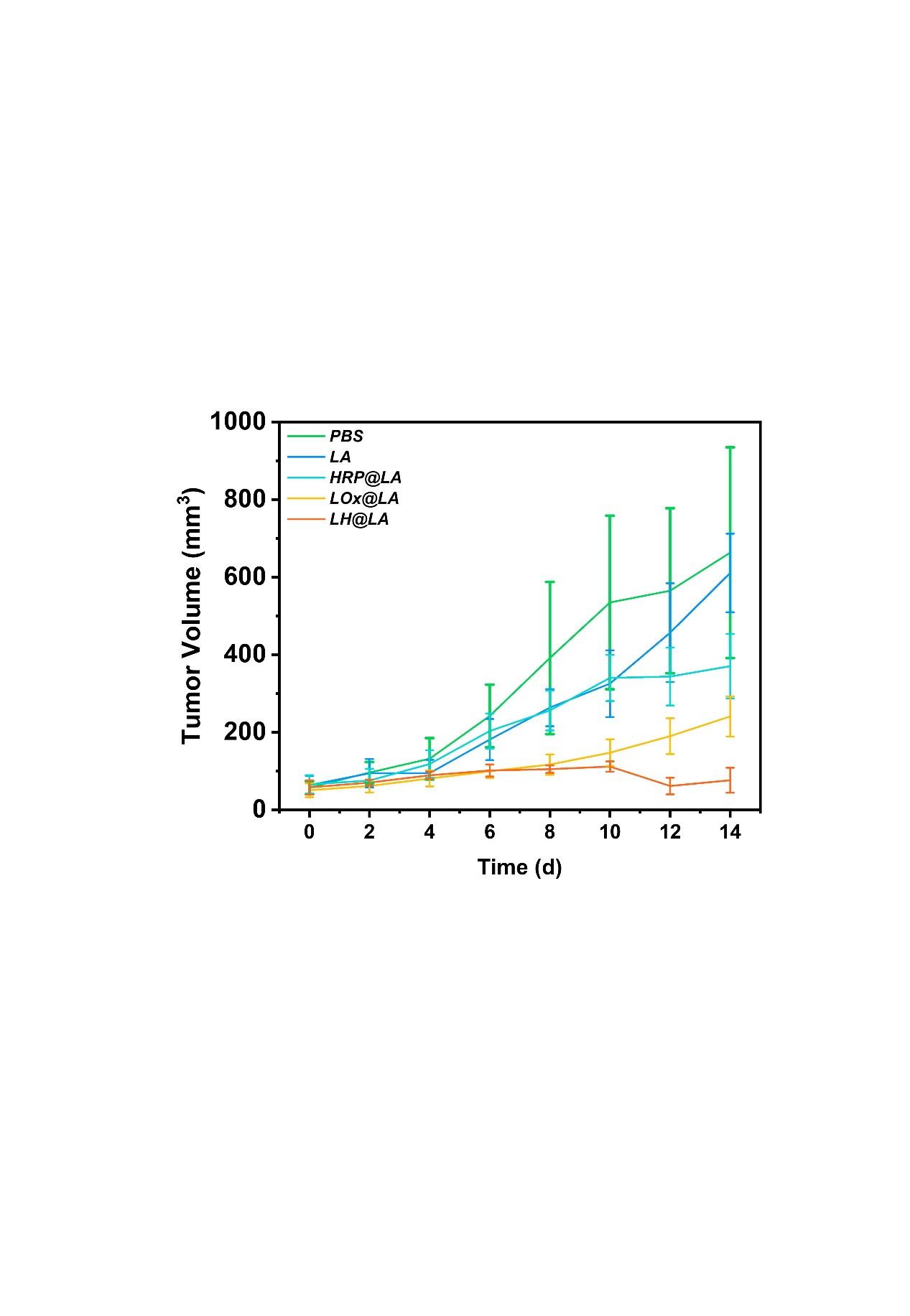


**Figure S18.** Tumor volume changes over time in different administration groups (n=5).


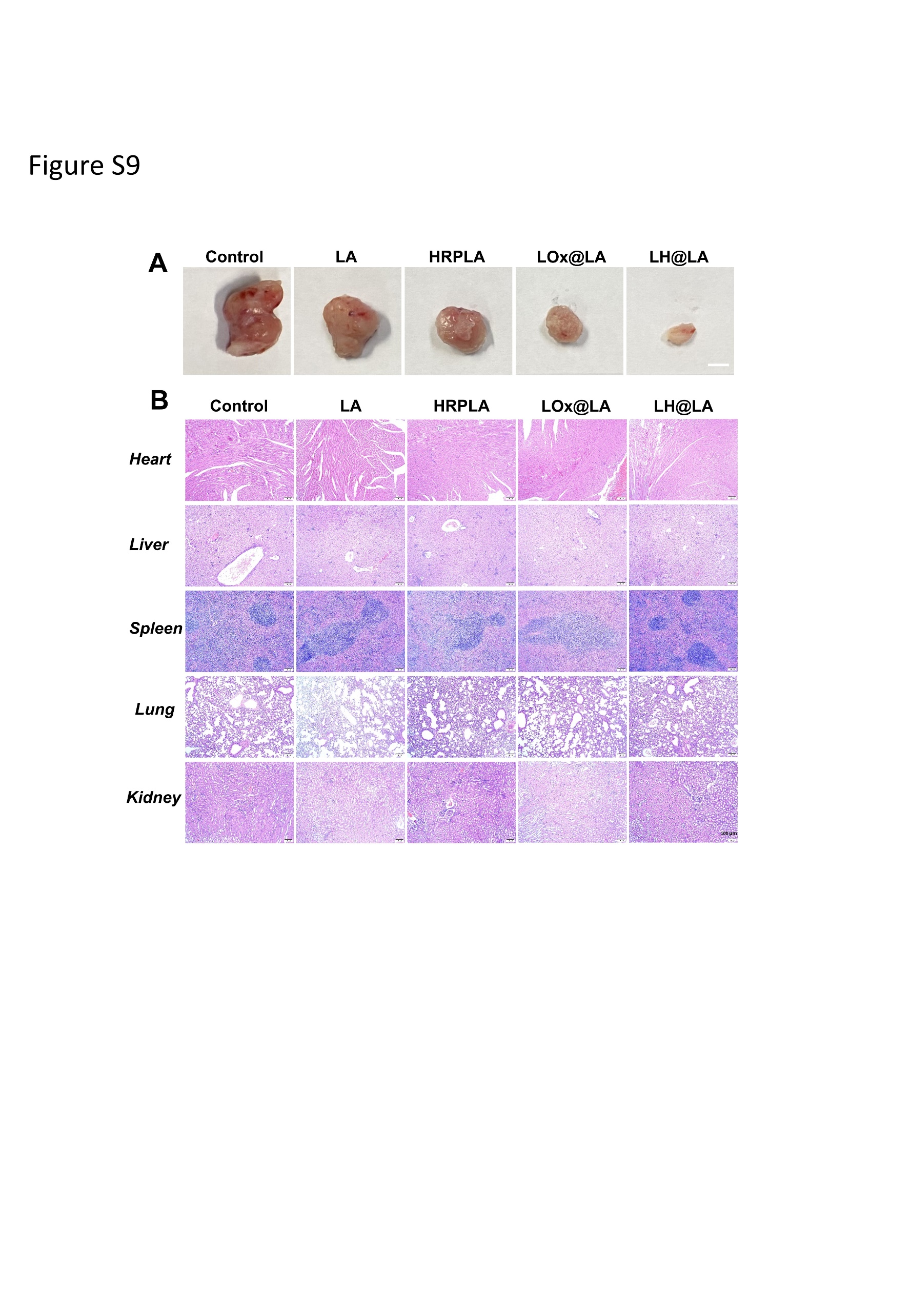


**Figure S19. A)** Tumor tissue image (Scale bar: 5 mm). **B)** H&E staining of major organ sections from different administration groups (Scale bar: 100 μm).

**
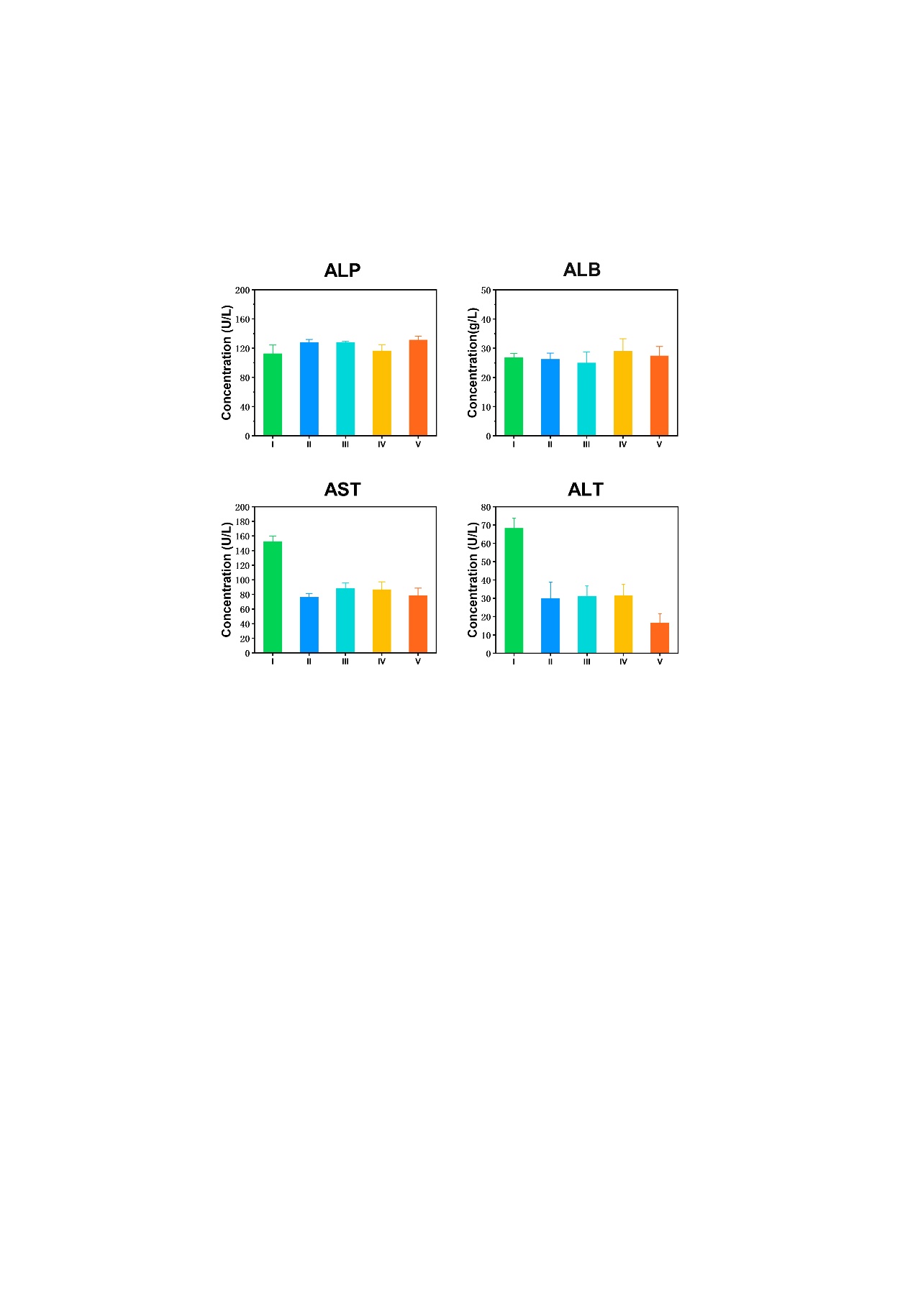
**

**Figure S20.** Concentration of ALP, ALB, AST and ALT in blood of different administration groups (n=3). (I: Control, II: LA, III: HRP@LA, IV: LOx@LA, V: LH@LA).

**
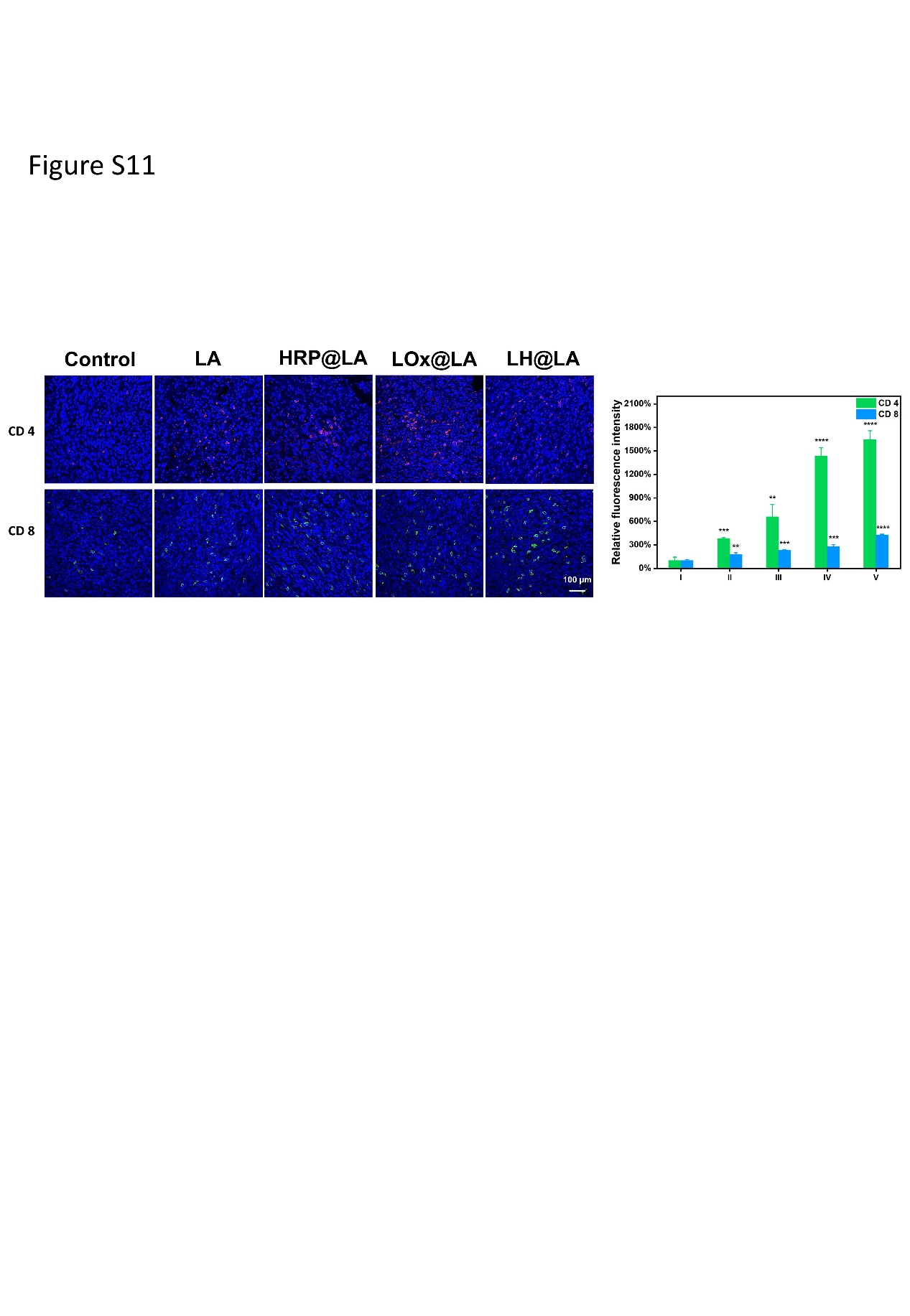
**

**Figure S21.** CD 4, CD 8 immunofluorescence staining of tumor tissue sections in different drug administration groups. (I: Control, II: LA, III: HRP@LA, IV: LOx@LA, V: LH@LA). Statistical significance was calculated by t-test for comparison between two groups. **P ≤ 0.01, ***P ≤ 0.001, ****P ≤ 0.0001.


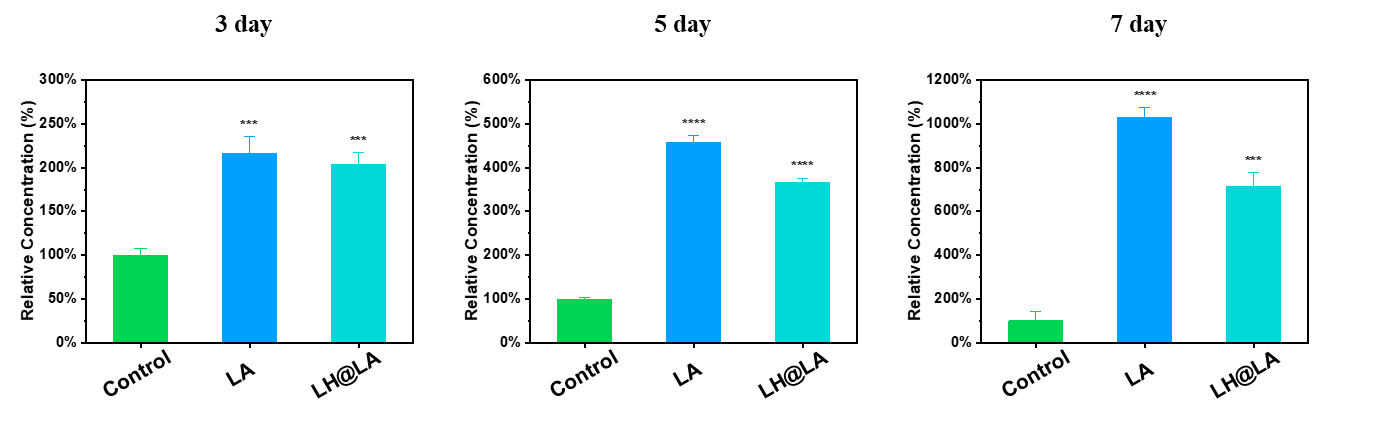


**Figure S22.** Detection of relative D-lactate concentration in tumor tissues at different time points after intra-tumoral administration of LH@LA.

**
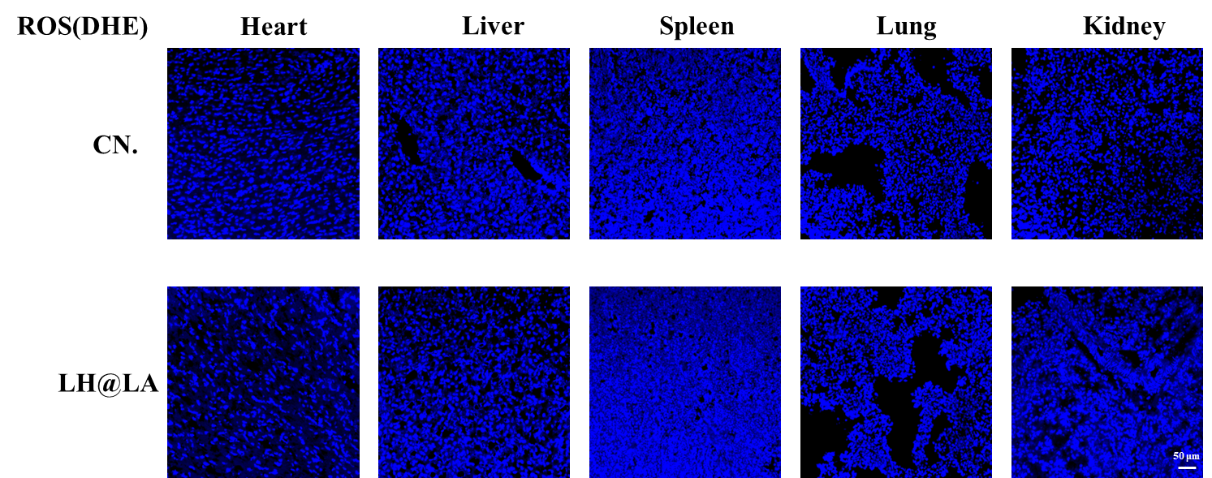
**

**Figure S23.** Fluorescence imaging of ROS staining in major organs of mice 14 days after intra-tumoral administration of LH@LA (Scale bar: 50 μm).

**
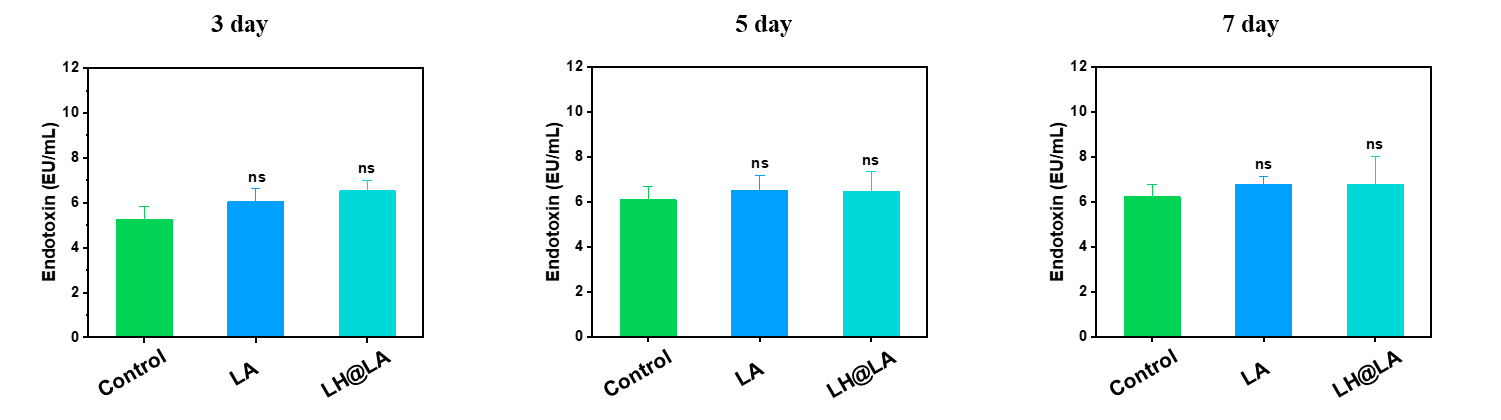
**

**Figure S24.** Detection of endotoxin concentration in mouse serum at different time points after intra-tumoral administration of LH@LA.
